# Bioinformatics Analysis of the Complete Genome Sequence of the Mango Tree Pathogen *Pseudomonas syringae* pv. syringae UMAF0158 Reveals Traits Relevant to Virulence and Epiphytic Lifestyle

**DOI:** 10.1371/journal.pone.0136101

**Published:** 2015-08-27

**Authors:** Pedro Manuel Martínez-García, Pablo Rodríguez-Palenzuela, Eva Arrebola, Víctor J. Carrión, José Antonio Gutiérrez-Barranquero, Alejandro Pérez-García, Cayo Ramos, Francisco M. Cazorla, Antonio de Vicente

**Affiliations:** 1 Centro de Biotecnología y Genómica de Plantas UPM-INIA, Parque Científico y Tecnológico de la Universidad Politécnica de Madrid. Pozuelo de Alarcón, Madrid, Spain; 2 Instituto de Hortofruticultura Subtropical y Mediterránea “La Mayora”, Universidad de Málaga, Consejo Superior de Investigaciones Científicas, Estación Experimental La Mayora, Algarrobo-Costa, Málaga, Spain; 3 Instituto de Hortofruticultura Subtropical y Mediterránea “La Mayora”, Universidad de Málaga, Consejo Superior de Investigaciones Científicas, Departamento de Microbiología, Facultad de Ciencias, Málaga, Spain; 4 Instituto de Hortofruticultura Subtropical y Mediterránea “La Mayora”, Universidad de Málaga, Consejo Superior de Investigaciones Científicas, Área de Genética, Facultad de Ciencias, Málaga, Spain; Virginia Tech, UNITED STATES

## Abstract

The genome sequence of more than 100 *Pseudomonas syringae* strains has been sequenced to date; however only few of them have been fully assembled, including *P*. *syringae* pv. syringae B728a. Different strains of pv. syringae cause different diseases and have different host specificities; so, UMAF0158 is a *P*. *syringae* pv. syringae strain related to B728a but instead of being a bean pathogen it causes apical necrosis of mango trees, and the two strains belong to different phylotypes of pv.syringae and clades of *P*. *syringae*. In this study we report the complete sequence and annotation of *P*. *syringae* pv. syringae UMAF0158 chromosome and plasmid pPSS158. A comparative analysis with the available sequenced genomes of other 25 *P*. *syringae* strains, both closed (the reference genomes DC3000, 1448A and B728a) and draft genomes was performed. The 5.8 Mb UMAF0158 chromosome has 59.3% GC content and comprises 5017 predicted protein-coding genes. Bioinformatics analysis revealed the presence of genes potentially implicated in the virulence and epiphytic fitness of this strain. We identified several genetic features, which are absent in B728a, that may explain the ability of UMAF0158 to colonize and infect mango trees: the mangotoxin biosynthetic operon *mbo*, a gene cluster for cellulose production, two different type III and two type VI secretion systems, and a particular T3SS effector repertoire. A mutant strain defective in the rhizobial-like T3SS Rhc showed no differences compared to wild-type during its interaction with host and non-host plants and worms. Here we report the first complete sequence of the chromosome of a pv. syringae strain pathogenic to a woody plant host. Our data also shed light on the genetic factors that possibly determine the pathogenic and epiphytic lifestyle of UMAF0158. This work provides the basis for further analysis on specific mechanisms that enable this strain to infect woody plants and for the functional analysis of host specificity in the *P*. *syringae* complex.

## Introduction

*Pseudomonas syringae* is a species of Gram-negative plant pathogenic bacteria that cause disease in many agriculturally important crops [1]. *P*. *syringae* infection provokes a variety of symptoms, such as leaf spots and blights, soft rots, stem knots, scabs or cankers, and leads to severe economic losses worldwide [2]. Based on plant pathogenicity test and host specificity, strains belonging to the *P*. *syringae* complex are subdivided into 57 pathovars [3]. In contrast, DNA hybridization segregated the *P*. *syringae* complex into at least nine different genomospecies [4,5] and multilocus sequence typing (MLST) permitted the classification into 13 phylogroups, including 23 differentiated clades [6]. Notably, the broad host range of the species as a whole contrasts with that of individual isolates, which can exhibit virulence potential in a diverse range or in a limited set of plant hosts [7,8], but the host range of each individual isolate frequently is not clear because experimental data about it are not available [9]. Moreover, many pathovars associated with unrelated plants are grouped together, sometimes even within the same clade [6]. Among plant pathogenic bacteria, *P*. *syringae* is an ideal system to study how evolutionary forces shape adaptation to different hosts, which makes it an archetype of plant-pathogen interactions [7,10].

To date, the complete genome or chromosome of at least 10 strains of *P*. *syringae* complex have been sequenced and fully assembled (http://www.ncbi.nlm.nih.gov/assembly/organism/136849/all/), including the three reference genomes, *P*. *syringae* pv. tomato DC3000 (DC3000), *P*. *syringae* pv. syringae B728a (B728a) and *P*. *syringae* pv. phaseolicola 1448A (1448A) (http://pseudomonas-syringae.org/) [11–13]. Additionally, more than 100 complete draft genomes of high quality are also available (http://www.ncbi.nlm.nih.gov/assembly/organism/136849/all/). DC3000, a pathogen that infects both tomato and *Arabidopsis*, is the causal agent of bacterial speck disease. In addition, both B728a and 1448A infect bean, but show significantly different phenotypes. B728a produces brown spot disease and exhibits an extensive epiphytic phase [12]. 1448A is the seed-borne causal agent of halo blight for bean, which is a calamitous disease in a number of first-world countries [13]. Many other *P*. *syringae* draft genome sequences exist for isolates from a great diversity of host plant [10]. These genomes exhibit drastic differences from each other, particularly in the presence/absence of virulence factors-associated genes [10]. Of these, the major determinants of pathogenesis include the effector proteins secreted through the type III secretion systems (T3SS) [14]. Once inside plants, effectors have the ability to promote virulence by disrupting and suppressing host immune signals. The super repertoire of effectors in the pangenome of the *P*. *syringae* species complex comprises 89 T3SS effector (T3E) proteins grouped into 64 families with DC3000 having the largest number of validated T3Es [10]. Furthermore, the host range of a given strain is thought to be mainly structured by the repertoire of the T3Es it encodes [14]. Which combination of effectors and other virulence-associated genes define the overall plasticity of host ranges is a question that remains unanswered.

*P*. *syringae* pv. syringae includes a diverse collection of strains isolated from different environments, many of them pathogenic on a variety of plant hosts, but showing different host range [6]. It also includes strains that cause mango tree apical necrosis, a disease affecting buds, leaves, and stems that has important economic impact worldwide [15]. Its pathogenic arsenal includes several T3Es and virulence factors, but the production of phytotoxins plays a crucial role during symptom development. Phytotoxins promote virulence by disrupting host metabolism and mimicking plant hormones [16]. Such is the case for mangotoxin, an antimetabolite toxin encoded by several pathovars of *P*. *syringae* genomospecies 1, which is produced in the early exponential growth phase and inhibits the enzyme ornithine *N*-acetyl transferase [17–19]. Production of mangotoxin, which has been observed in almost all of the *P*. *syringae* pv. syringae strains isolated from mango tissues, requires involvement of the *mgo* and *mbo* operons, whose contribution to virulence has been mostly analyzed in the model strain UMAF0158 [18–21]. Interestingly, phylogenetic analyses grouped *P*. *syringae* pv. syringae strains isolated from mango with mangotoxin-producing strains isolated from other plants [22]. Unlike herbaceous hosts, mango trees provide infection and overwintering sites, which are unique to woody perennials. This characteristic is reflected in the lifestyle of mango-associated *P*. *syringae* strains and the way they induce disease symptoms in plants [23]. In this regard, genome sequencing and comparative genomics are useful tools for the identification of genetic elements of *P*. *syringae* strains that enable the colonization of woody plants as olive, horse chestnut, plum, maple tree or kiwi [10,24–26], which will provide insight into the interactions of *P*. *syringae* and woody plants.

Here, we report the complete sequence, annotation and bioinformatic analysis of the *P*. *syringae* pv. syringae UMAF0158 genome (chromosome and pPSS158 plasmid), highlighting the virulence and plant interaction genetic background of this pathogenic bacteria on mango trees. We also compared its genome with those of other 25 sequenced *P*. *syringae* strains from the *P*. *syringae* complex with a special focus on *P*. *syringae* pv. syringae B728a, which shares approximately 91% of the UMAF1058 protein coding genes, but they grouped into different pv. syringae phylotypes [22] and clades of phylogroup 2 [6]. Our analysis highlights genetic differences between these two strains, which may confer UMAF0158 its ability to infect mango trees. In addition to the presence of the mangotoxin biosynthetic operon *mbo*, the UMAF0158 genome differs from that of B728a in the codification of a cellulose synthase operon and in harboring two additional secretion systems, i.e., a T3SS and a T6SS. Moreover, UMAF0158 displays a different repertoire of T3Es, which may be a determinant of its association with mango trees. Our data provide the basis for further functional studies of the virulence mechanisms and host specificity determinants of the *P*. *syringae* pv. syringae UMAF1058, a representative strain of phylotype 1 of this pathovar and the clade 2a of phylogroup 2 of the *P*. *syryngae* complex, which is pathogenic on a woody plant and whose complete genome sequence has been made available.

## Materials and Methods

### Bacterial Growth and DNA methods

The bacterial strain UMAF0158 (CECT 7752) belonging to *Pseudomonas syringae* pv. syringae and derivative mutants were routinely grown in KB medium with 48 h of incubation at 28°C for further studies.

Ten colonies from a pure culture of UMAF0158 strain were inoculated on 100 ml of LB medium and grown for 15 h at 28°C with shaking (150 r.p.m.). After this period, the OD_600nm_ of the culture was 1.8. Serial dilutions of this culture and plating on LB plates yielded 1.3×10^9^ CFU/ml of pure bacterial culture. The rest of the culture was divided into 54 aliquots of 1.5 ml, and DNA was extracted from all of the cultures using the *Jet-Flex genomic DNA purification* kit (Genomed GmbH, Germany). DNA samples were collected together and further purified by extraction with 1:1 phenol:chloroform and precipitated with 4 M NaCl and 13% PEG. DNA was suspended in 500 μl MilliQ H_2_O, and *NanoDrop* measurements indicated 3.6 μg/μl (in total 1800 μg of DNA) with an A_260_/A_280_ of 1.85. The extracted DNA was visualized in 1% agarose after digestion with the restriction enzymes *Eco*RI and *Pst*I.

### Whole Genome Sequencing

The finished UMAF1058 genome was generated at the Beijing Genomics Institute (BGI-HK) using an Illumina HiSeq 2500 system. Briefly, the isolated DNA was used to generate three libraries of 500 bp, 2000 bp and 6000 bp, producing 1336, 1312 and 1352 Mb of raw data, respectively. These were passed through a filtering pipeline that removed known sequencing and library preparation artifacts. After data treatment, the SOAPdenovo 1.05 software package [27,28] was used for sequence assembly and quality assessment. Assembly results were then combined and mapped to the genome of *P*. *syringae* B728a, yielding to two scaffolds corresponding to one chromosome and one plasmid. Finally, a PCR gap closure and three circle PCR verification were performed to obtain the final complete sequence of chromosome and pPSS158 plasmid. Further details of the whole process are described in S1 File.

### Genomic data and annotation

The assembled genome of UMAF0158 was submitted to the NCBI Prokaryotic Genome Annotation Pipeline for automatic annotation and manually reviewed. Gene locations and protein products were generated from above annotation (ASN.1 file) using the script “asn2all” belonging to the NCBI ToolKit (http://www.ncbi.nlm.nih.gov/toolkit). Genome sequences (DNA, proteins and gene locations) of DC3000, B728a and 1448A were downloaded from the NCBI complete bacterial genome repository (ftp://ftp.ncbi.nlm.nih.gov/genomes/Bacteria), while the corresponding sequences of *P*. *savastanoi* pv. savastanoi strain NCPPB3335 were downloaded from ASAP (https://asap.genetics.wisc.edu/asap/home.php; [29]. The rest of the *P*. *syringae* genome sequences (DNA, proteins and gene locations) were downloaded from the NCBI draft bacterial genome repository (ftp://ftp.ncbi.nlm.nih.gov/genomes/Bacteria_DRAFT/). Proteins and gene locations were also generated from DNA sequences (fna files) using Glimmer v3.02 [30] and only very subtle differences were obtained. All genomes were downloaded on July 15, 2014. The accession numbers and references for all the genome sequence data used in this work are summarized in S1 Table.

The UMAF0158 annotation of COGs was performed by aligning the set of predicted protein sequences against the COG PSSM of the CDD (http://www.ncbi.nlm.nih.gov/cdd) using rpsblast. Hits with an *E*-value ≤ 0.001 were first retained. Then, only the best hit was selected for each protein. The same procedure was used to assign COG categories to the repertoire of predicted proteins of the B728a, DC3000 and 1448A strains. Predictions of horizontally transferred regions and prophages were computed using Alien Hunter 1.7 [31] and Prophage Finder [32], respectively. T346Hunter [33] was used to identify secretion systems clusters.

### Trinucleotide composition

The distribution of all 64 trinucleotides was determined for the whole chromosome and 2 kb sub-windows. Then, the χ^2^ statistic of the difference between the trinucleotide composition of each window and that of the whole chromosome was computed. Large values for χ^2^ in a given window denote different trinucleotide compositions from the rest of the chromosome. Probability values were computed assuming uniform distribution of the DNA composition along the genome. Because this assumption may have been incorrect, high χ^2^ values were interpreted as indicators of unusual regions on the chromosome that require further investigation.

### Protein and DNA-based phylogenetic trees

Phylogenetic analyses were performed by multilocus sequence analysis using a concatenated dataset for *gapA*, *gltA*, *recA*, *rpoA* and *rpoB*. Phylogenies trees were obtained using the Maximum Likelihood method based on the JTT (Jones-Taylor-Thornton) matrix-based model [34]. The percentage of trees in which associated taxa clustered in the bootstrap test (1000 replicates) is shown next to the branches in the corresponding Figures [35]. Multiple alignments and evolutionary analyses were conducted using MEGA5 software [36].

### Comparative Genomics

Each of the predicted proteins in UMAF0158 was compared to those of the other *P*. *syringae* strains using BLASTP (*E*-value ≤ 1×10^−10^). The same procedure was used to compare predicted proteins in B728a with those in UMAF0158.

### Distribution of T3Es

We performed BLASTP searches of the T3Es in http://pseudomonas-syringae.org/ against the 26 *P*. *syringae* proteomes. First, only hits with an *E*-value ≤ 1×10^−10^ were retained. If no hits were found for a given T3E, it was considered absent. For a given strain, when a gene product was found to match with several T3Es, the one with the best *E*-value was selected. If there were more than one T3E with the best *E*-value, the alignment with the greatest number of identities was retained. Then, lengths of the query T3Es were compared to those of the alignments. We labelled a potential T3E as incomplete when the alignment was ≥ 25% smaller than the length of the original T3E. Otherwise, the T3E was labelled as complete. Based on the presence of complete and incomplete T3Es, a matrix was created and used to generate a dendrogram by means of the R package APE [37]. Further information on this analysis can be found in S2 Table.

### Circular Genome Visualization

Circular layouts were generated using Circos [38].

### Accession Numbers

The finished genomic sequences of UMAF0158 have been deposited in GenBank under accession numbers CP005970 (chromosome) and CP005971 (plasmid pPSS158).

### Laboratory procedures

#### Construction of T3SS Mutants

Single mutants in the orthologous genes of the T3SS (*hrp* cluster) and a second rhizobial-like T3SS-2 (cluster *rhc*) and double mutants in both T3SSs were constructed in UMAF0158, using the protocol described by Zumaquero et al [39]; the used primers are summarized in S3 Table. Additionally, the simple mutant in T3SS-1 (UMAF0158Δ*hrpL*) was complemented using the replicative vector pBBR1 MCS-5 [20] containing the *hrpL* gene. The primers used to confirm the integration were as follows: hrpL_HindIII_for (TTaaGCTtGCATGGTTATCGC) and hrpL_XbaI_rev (CGTtCtAGaTGGTTCCAGAC).

#### Bacterial motility

Swimming and swarming motility were tested *in vitro* using KB medium diluted at 20%, and using a 0.3 and 0.5% agar concentration to determine both motility styles. All strains used in this experiment were inoculated in the center of the plate and incubated at 28°C for 24 h while being careful to maintain the plates completely horizontal. The diameter of displacement was measured in all plates. The experiment was performed three times with five replicates for each.

#### Toxicity in worms

To further investigate the toxicity of the parental strain and defective derivative mutants, both simple and double mutants were tested in the *Caenorhabditis elegans* model system. The worms were in the same larval stage for when egg preparation was complete. Afterward, the eggs were incubated on *Escherichia coli* OP50 feeding plates at 20°C for approximately 76 h. At the time point the egg preparations were made, 5 ml of LB liquid media was incubated with the experimental strains used, which were previously incubated for 48 h at 28°C. The day after, 100 μl of the bacterial culture was added onto 6-well plates and incubated again overnight at 30°C until a bacteria lawn formed. On the next day, the synchronized worms were washed away from the feeding plates with M9 buffer and transferred to the bacterial lawns in the 6-well plates (20–40 worms per well). The worms were counted after 24, 48, 72 and 96 h with the aid of a bifocal magnifier [40].

A complementary toxicity study was performed with *Galleria mellonella*. In this experiment, UMAF0158 parental and simples and double T3SS mutants diluted 1:100 were inoculated from an overnight culture in new liquid LB culture. When the OD was between 0.4 and 0.9, 10 ml was harvested and centrifuged at room temperature at 4000 rpm for 10–15 min. The pellets were resuspended in 10 ml 10 mM MgSO_4_7H_2_O to an OD value of 1. From these stocks, a dilution series were prepared by 50% dilutions, and 10 μl of every dilution was injected into of each *G*. *mellonella* larva. Five larvae per bacteria were inoculated, using 10 mM MgSO_4_7H_2_O as a negative control. The larvae were maintained in a Petri dish at 30°C, and the evaluation of the health state was checked after 20, 24, 48 and 72 h. Both toxicity tests were performed twice.

#### Adhesion in mango leaf

To determine the adhesive ability to the mango leaf surfaces (*Mangifera indica* L. var. Osteen), wild type and derivative mutants were grown in KB medium for 48 h at 28°C, and the final OD was adjusted to approximately 10^8^ CFU/ml (0.7–0.8 at 600 nm). Drops of 10 μl for each strain were placed on the same clean mango leaf to which it had painted dividing lines to avoid mixture. The two years old mango plants were maintained in a conditioned room, with adjustable temperature to 25°C and light cycles of 16h, environmental relative humidity was around 70–80%. After 30 min, the leaf was carefully washed by sterile water, cut from a mango tree and cut again by lines for processing. Leaf pieces were placed into sterile bags with 1 ml of sterile water and homogenized for 3 min using a lab blender. Serial dilutions of 100 μl from homogenized tissues were plated in KB medium to count recovered cells. Three plates per strain were used, and three replicates per experiment and three independent experiments were performed to obtain the adhesion experiments results. Statistical analysis were performed with IBM.SSPS 19 software (IBM Company, Armonk, NY) using ANOVA of one factor for analysis of the means with *P* = 0.05.

#### Survival on tomato plant surfaces

Bacterial suspensions of UMAF0158 and defective mutants adjusted to 10^8^ CFU/ml were used to spray 6-week-old tomato plants. Two inoculated tomato plants per strain and a control (sterile water) were used. The tomato plants (*Solanum lycopersicum* L. cv. Hellfrucht-Früstamm) of two weeks old were maintained in a conditioned room in a 16 h photoperiod at 25°C. Two leaflets of each plant were taken three times a week for 21 days for analysis. The leaflets were divided into two pieces using a sterile scalpel and cut along the midrib. One half was promptly processed, and the second half was processed after disinfection using 3% hydrogen peroxide for 5 min and washed in sterile water for another 5 min. The samples were homogenized in 1 ml sterile water, diluted in a decimal series and plated in rich medium. Colonies were counted after two days of incubation at 28°C. Two independent experiments were performed and three replicates were included in each experiment.

#### Hypersensitivity reaction in tobacco

To determine hypersensitivity reaction or cell death in tobacco leaves, bacterial suspensions of wild type UMAF0158 and defective mutants were adjusted to 10^6^ CFU/ml. Infiltrations in tobacco leaves at 10^6^, 10^5^, 10^4^ and 10^3^ CFU/ml were performed. Cell death was checked after 3 and 7 days.

#### Pathogenesis on tomato leaves

Inoculations on detached tomato leaflets were conducted by wild type UMAF0158 and mutant derivatives. The evaluation of the pathogenesis was performed according to previous evaluations made with the wild type UMAF0158 strain [18,41]. Bacterial suspensions from exponentially growing cultures were adjusted to 10^8^ CFU/ml. Detached leaflets were inoculated by placing six 10 μl drops of the bacterial suspension on six different places on each leaflet. Inoculations were then performed by piercing through the droplets with a sterile entomological pin. The leaflets were maintained in Murashige & Skoog (MS) media at 22°C for a 16 h photoperiod. Six tomato leaflets were used for each strain and independent experiment. Non-infected detached leaflets inoculated with sterile distilled water were included in all experiments as a control. These experiments were repeated three times.

The development of necrotic symptoms at the inoculation points was determined every day for 10 days. The appearance of necrotic symptoms was monitored by visual analysis to evaluate disease incidence (number of inoculated points with symptoms of necrosis), considering points with areas between 0.2 and 0.5 cm (Category 2) equal or greater than 0.5 cm (Category 3) in diameter. Severity analysis was performed by image analysis with Visilog 5.0 software of the total necrotic area developed per leaflet on the last day of the assay. Statistical analysis of incidence was performed using SAS9.2 software (SAS Institute Inc., Cary, NC, USA) with Enterprise Guide 4.2 using generalized linear model analysis. Severity results were analyzed by one way ANOVA by IBM.SSPS 19 software (IBM Company, Armonk, NY).

## Results and Discussion

### General features

**Fig 1:**
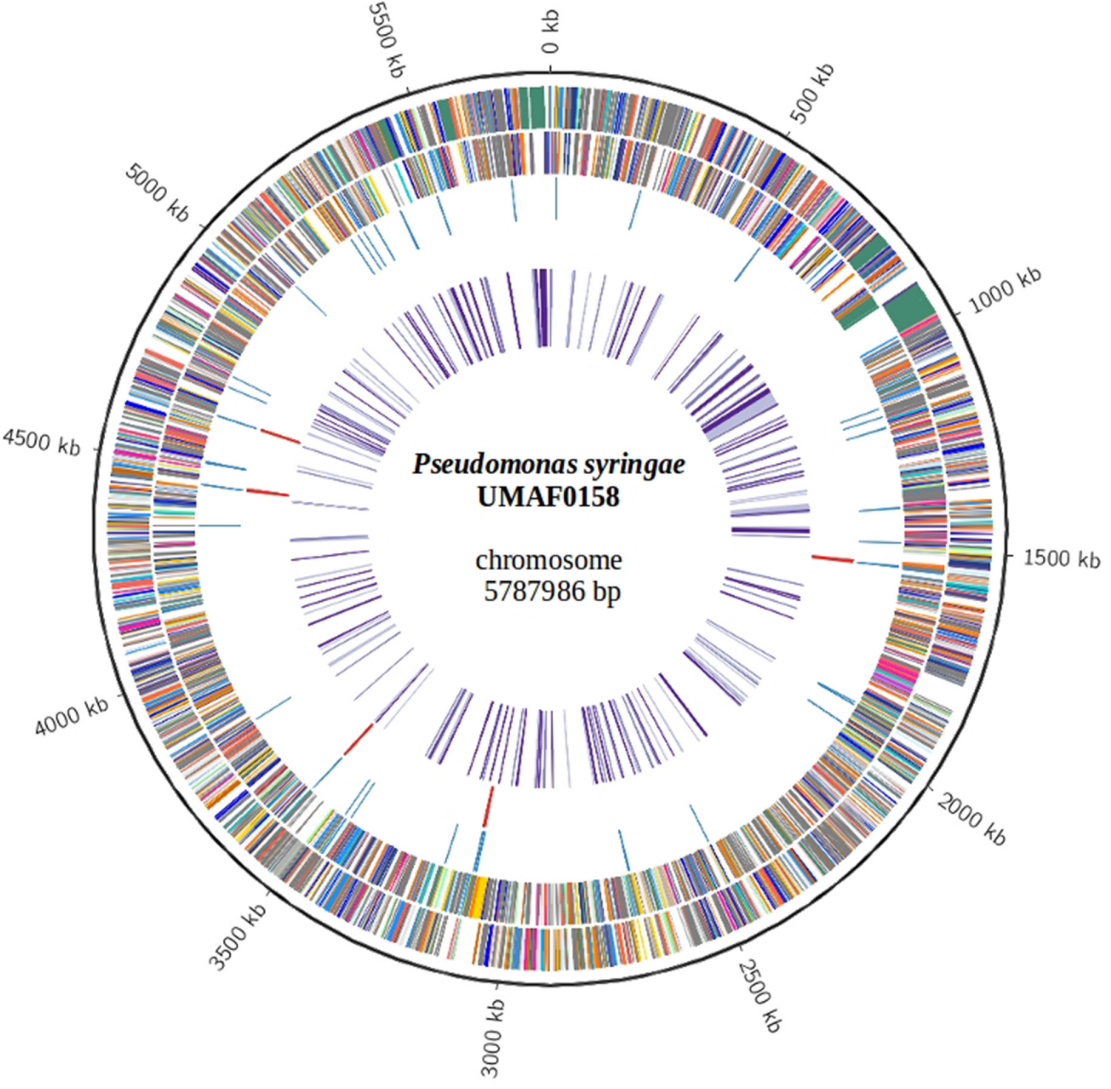
Features of the *Pseudomonas syringae* pv. syringae UMAF0158 chromosome. From the outside in, the outermost circle (black) shows the scale line; circles 2 and 3 represent predicted coding regions on the plus and minus strand, respectively, which are color coded based on COG categories; circles 4 and 5 show tRNA (blue) and rRNA (red), respectively; circle 6 depicts ORFs associated with virulence (purple).

The *P*. *syringae* pv. syringae UMAF0158 genome is composed of one circular chromosome of 5787986 bp (Table 1; Fig 1) and one plasmid. The pPSS158 plasmid (GenBank CP005971) of 63004 bp has an average GC content of 54.6% and 71 coding sequences (CDSs) (Table 1), where is remarkable the presence of characteristic genetic traits for conjugative plasmids of the pPT23A-like family, such as *repA*, a T4SS conjugative system and the *rulAB* genes [42]. Conjugative plasmids harboring *rulAB* genes contributes to UV and solar radiations tolerance and epiphytic fitness, as it was previously demonstrated [42]. However, most of CDSs found on the plasmid pPSS158 were annotated as hypothetical proteins and any potentially gene clearly associated with virulence has been determined (S4 Table). In total, 5017 CDSs were identified within the UMAF0158 chromosome, which has an average GC content of 59.3% (Table 1). Among the predicted chromosomal CDSs, a putative function was assigned to 4030 (80%), while the remaining 987 CDSs were designated as hypothetical proteins. A total of 18 genes were predicted to be pseudogenes. The classification of the UMAF0158 CDSs into functional categories according to the COG (Clusters of Orthologous Groups) database is summarized in Table 2 in comparison with *P*. *syringae* pv. syringae B728a, *P*. *syringae* pv. tomato DC3000 and *P*. *syringae* pv. phaseolicola 1448A. With the exception of category L (replication, recombination and repair), which includes a reduced number of UMAF0158 CDSs (137) in comparison with those of the other three genomes (187, 272 and 370 CDSs for B728a, 1448A and DC3000, respectively), no significant differences were found regarding the remaining functional categories, further supporting the relatedness of these four strains. In addition to CDSs, a total of 63 tRNAs and five rRNA operons were found on the UMAF0158 chromosome.

**Table 1:** General features of the *Pseudomonas syringae* pv. syringae UMAF0158 genome and comparison with *P*. *syringae* pv. syringae B728a, *P*. *syringae* pv. phaseolicola 1448A and *P*. *syringae* pv. tomato DC3000.

Feature	PssUMAF0158		PssB728a	Psp1448A			PstDC3000		
Molecule	Chromosome	pPSS158	Chromosome	Chromosome	p1448A-A	p1448A-B	Chromosome	pDC3000A	pDC3000B
Size (bp)	5787986	63004	6093698	5928787	131950	51711	6397126	73661	67473
G+C content (%)	59.3	54.6	59.2	58.0	54.1	56.0	58.4	55.1	56.1
CDSs predicted no.	5017	71	5089^a^	4985^a^	127*	60^a^	5481^a^	68^a^	70^a^
No. of rRNAs	16	-	16	16	-	-	15	-	-
No. of tRNAs	63	-	64	64	-	-	63	-	-
Reference	This study		[12]^a^	[13]^a^			[11]^a^		

^a^ The number of predicted CDSs corresponds to those indicated at NCBI for the corresponding genome sequences (March 1st, 2015).

**Table 2:** Number of CDSs associated with COG functional categories in the *Pseudomonas syringae* pv. syringae UMAF0158 genome and comparison with *P*. *syringae* pv. syringae B728a, *P*. *syringae* pv. phaseolicola 1448A and *P*. *syringae* pv. tomato DC3000.

	Funtional Category	UMAF0158	B728a	1448A	DC3000
A	RNA processing and modification	1	1	1	1
B	Chromatin structure	1	1	1	1
C	Energy production and conversion	223	231	210	234
D	Cell cycle control, cell division, chromosome partitioning	38	46	41	44
E	Amino acid transport and metabolism	459	467	458	461
F	Nucleotide transport and metabolism	89	87	88	81
G	Carbohydrate transport and metabolism	276	268	263	264
H	Coenzyme transport and metabolism	177	178	179	173
I	Lipid transport and metabolism	163	162	166	176
J	Translation, ribosomal structure and biogenesis	198	205	204	201
K	Transcription	360	360	343	367
L	Replication, recombination and repair	137	187	272	370
M	Cell wall/membrane/envelope biogenesis	270	288	270	263
N	Cell motility	163	166	168	160
O	Posttranslational modification, protein turnover, chaperones	156	157	152	157
P	Inorganic ion transport and metabolism	272	277	287	278
Q	Secondary metabolites biosynthesis, transport, and catabolism	119	128	112	118
R	General function prediction only	521	522	520	544
S	Function unknown	385	393	356	406
T	Signal transduction mechanisms	343	349	339	358
U	Intracellular trafficking, secretion, and vesicular transport	150	148	156	136
V	Defense mechanisms	48	53	48	58
Z	Cytoskeleton	1	1	1	0
	Total	4550	4675	4635	4851

### Phylogeny

**Fig 2:**
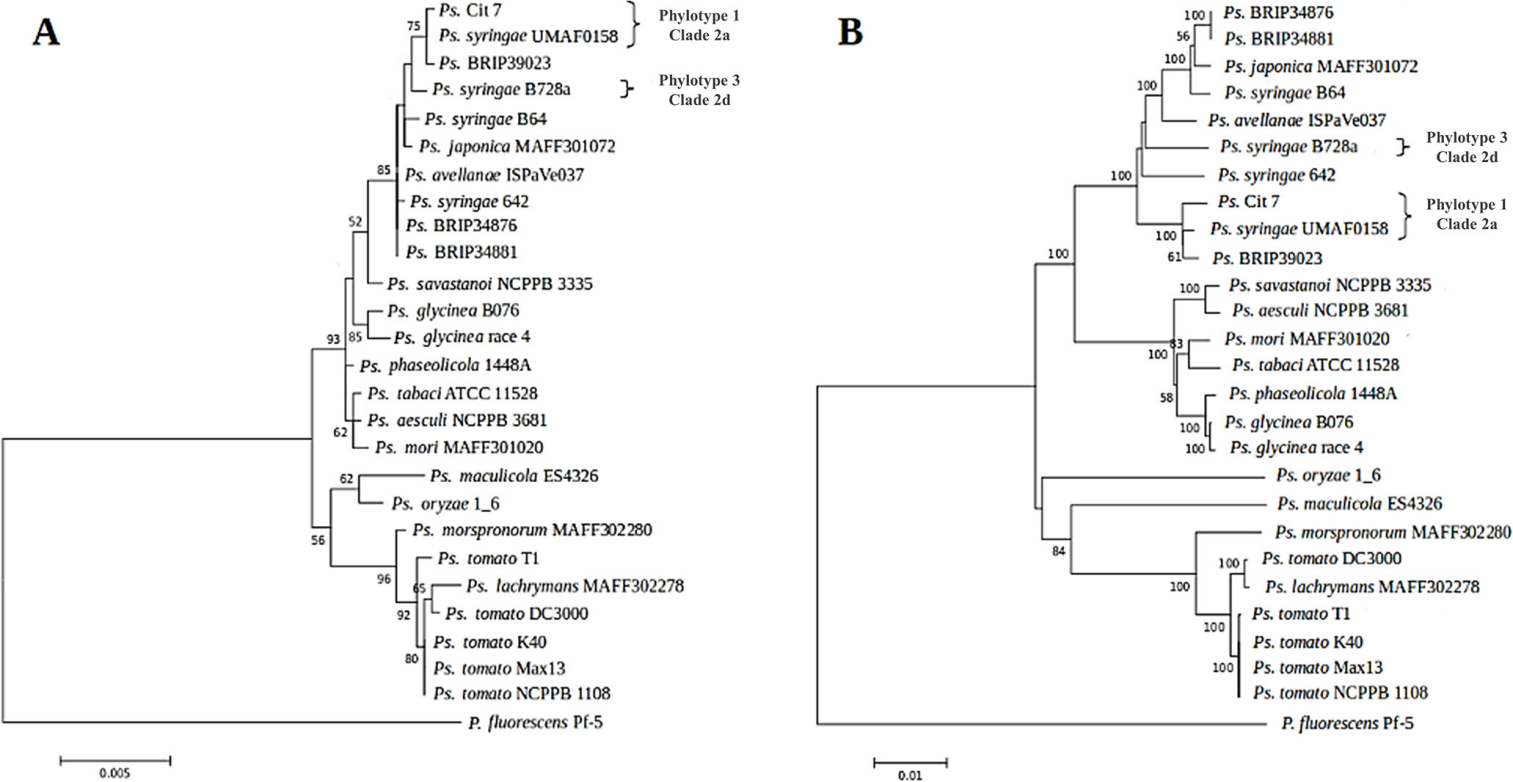
Phylogenetic analyses of *Pseudomonas syringae* pv. syringae UMAF0158 and 25 selected strains of the *P*. *syringae* complex (see S1 Table). Multilocus sequence analysis were performed using a concatenated dataset for *gapA*, *gltA*, *recA*, *rpoA* and *rpoB*. The evolutionary history was inferred using the Maximum Likelihood method based on the JTT matrix-based model. The percentage of trees in which the associated taxa clustered in the bootstrap test (1000 replicates) is shown next to the branches. *P*. *fluorescens* strain Pf-5 was used as an outgroup. **A,** Phylogeny based on protein products. **B,** Phylogeny based on DNA sequences. Some strains were labelled with the corresponding phylotype of pv. syringae [21, 22] and clade of phylogroup 2 of *P*. *syringae* complex [6]. The alignments used to generate this figure have been included as supporting information (S2 File).

In order to establish the phylogenetic relationship between UMAF0158 and other related *P*. *syringae* strains, we selected 25 genome sequenced strains (S1 Table) and compared a set of five protein-coding house-keeping genes, namely *gapA*, *gltA*, *recA*, *rpoA* and *rpoB*. We created an alignment of the proteins and reconstructed the phylogenetic tree shown in Fig 2, using neighbor-joining methods. The strain *P*. *fluorescens* Pf-5 was used as outgroup. The resulting phylogeny clustered UMAF0158 with *P*. *syringae* Cit 7, a strain originally isolated from a healthy orange tree [10,43], and more separate from B728a, the model strain for pv. syringae. This result is in agreement with previous phylogenetic analyses, which clustered both UMAF0158 and Cit 7 in phylotype 1 [21,22]. Such a phylotype of the pathovar syringae is mainly associated with the mango host and characterized by mangotoxin production [22]. Additionally, UMAF0158 and other strains of phylotype 1 are pathogenic on mango, lilac, tomato or pear [15], but not on bean, in contrast with B728a, which is pathogenic on bean but showed low virulence on mango [12,22]. Given that UMAF0158 is the only strain belonging to this group whose complete genome is available, it could be taken as a representative of phylotype 1 for pv. syringae [22] and clade 2a of phylogroup 2 of *P*. *syringae* complex [6].

Regarding the other *P*. *syringae* strains in the phylogeny with complete genome sequences, B728a was the closest to UMAF0158. Accordingly, this strain shares the highest number of CDSs predicted in UMAF0158 (see next section). These data, together with the fact that both B728a and UMAF0158 belong to *P*. *syringae* pv. syringae, prompted us to pay special attention to the genomic differences between these two strains.

### Comparative genomics

**Fig 3:**
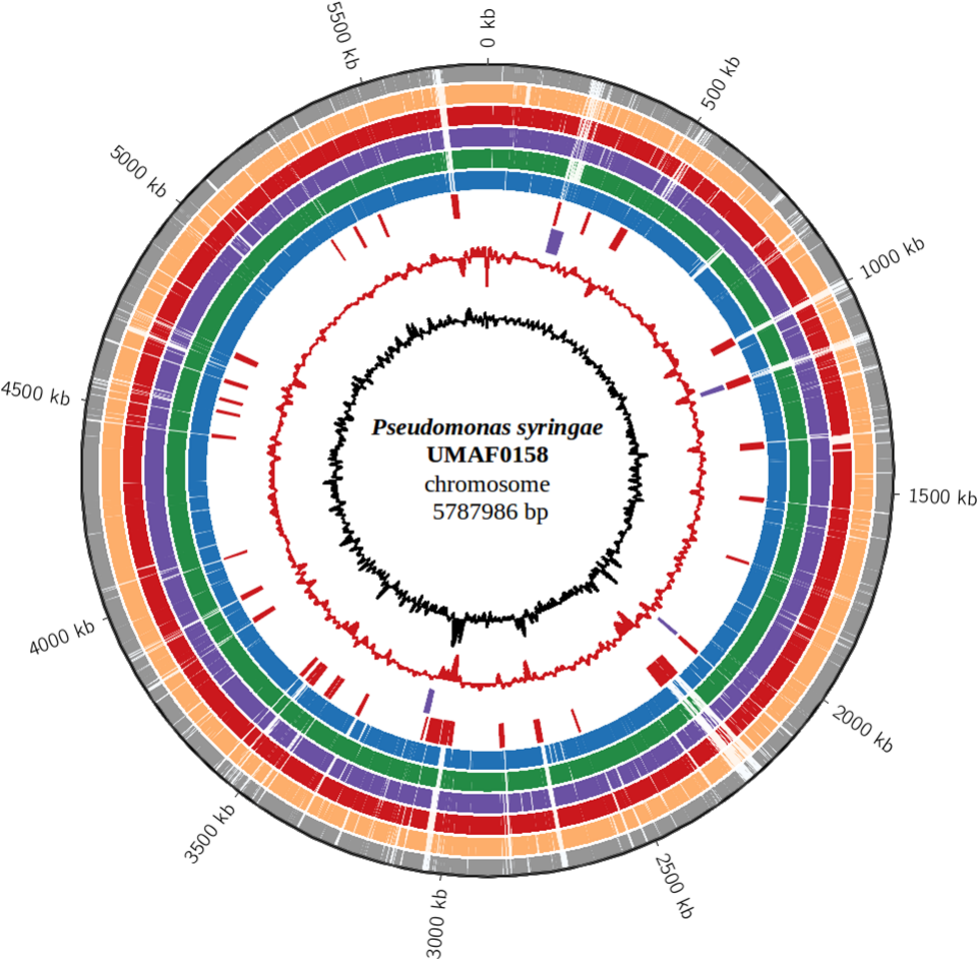
Conservation analysis of the *Pseudomonas syringae* pv. syringae UMAF0158 chromosome. From the outside in, the outermost circle (black) shows the scale line. Circles 2 to 4 display similarity (*E*-value ≤ 1×10^−10^) among UMAF0158 and the three *P*. *syringae* with complete genome sequences: DC3000 (grey), 1448A (orange) and B728a (red). Circles 5 to 7 display similarity (*E*-value ≤ 1×10^−10^) among UMAF0158 and the draft genomes of the three phylogenetically closest *P*. *syringae* strains among the 25 selected in this study: 642 (purple), BRIP39023 (green) and Cit 7 (blue). Circles 8 and 9 display putative horizontally transferred regions (red) and prophages (purple), respectively; circle 10 shows G+C in relation to the mean G+C in 2 kb windows (red); circle 11 shows trinucleotide composition (black).

The sequence of the UMAF0158 chromosome was compared to that of selected *P*. *syringae* strains (Fig 3). Of the 5017 CDSs predicted in UMAF0158, 4912 (98%) have orthologs (BLASTP *E*-value ≤ 1×10^−10^) in other *P*. *syringae* and 3570 (71%) are present in all strains. All of the 105 genes found to be unique to UMAF0158 are heavily enriched in hypothetical proteins (103). The other two genes include a membrane protein (PSYRMG_17725) and a flavodoxin (PSYRMG_09680).

Among the selected strains, Cit 7, BRIP39023 and 642 share the highest number of CDSs compared to UMAF0158. These three strains, which belong to pv. syringae or are closed to it and whose complete genome sequences are not yet available, share 93.6, 93.4 and 91.2% of the CDSs predicted in UMAF0158, respectively (Fig 3). Regarding *P*. *syringae* with complete genome sequences, B728a shares 90.7% of the CDSs followed by 1448A and DC3000, which share 88.4 and 88.1%, respectively (Fig 3).

Fig 3 shows some sequence features associated with mechanisms of horizontal transfer, including regions with differential distributions of trinucleotides and GC-content, predicted prophages and putative horizontally transferred genes. In most cases, these features match with non-conserved regions of the UMAF0158 chromosome (white-colored in the six most outer rings of Fig 3 *)*.

### Comparison of CDSs between UMAF0158 and B728a

In order to compare the genomes of UMAF0158 and B728a, we proceeded to identify regions enriched in coding genes in either strain that are not present in the other. The search was performed so that only regions spanning at least 4000 bp were retained (the whole set of differential protein coding genes are listed in S5 and S6 Tables). These regions are summarized in Tables 3 and 4. Thirteen regions were identified in UMAF0158 with sizes ranging from 6051 to 20822 bp. Eight of such regions are highly enriched in hypothetical proteins (at least 80% of their CDSs). Two of the remaining five regions contain a combination of mobile genetic elements and hypothetical proteins. The remaining regions correspond to three operons: an additional rhizobial-like T3SS Rhc (PSYRMG_02470–02585), which is analyzed below (S1 Fig), a cellulose production operon (PSYRMG_20805–20845), and the well-described mangotoxin biosynthetic operon *mbo* [19,21] (PSYRMG_10110–10135). This operon is present in only a limited number of strains belonging to genomospecies 1, and it has been acquired once during evolution by horizontal transfer [21,22].

**Table 3:** Regions of *Pseudomonas syringae* pv. syringae UMAF0158 genome with low similarity to *P*. *syringae* pv. syringae B728a.

Location (bp)	Length	No. of CDSs	No. of hypothetical	No. of CDSs not present in B728a	Relevant features
248304–257525	9221 bp	16	9 (56%)	15 (94%)	mobile genetic elements
258593–272300	13707 bp	21	11 (52%)	19 (90%)	mobile genetic elements
519318–537403	18085 bp	20	8 (40%)	15 (75%)	T3 secretion components
996632–1011913	15281 bp	17	17 (100%)	17 (100%)	hypothetical proteins
1133844–1143487	9643 bp	20	19 (95%)	18 (90%)	mobile genetic elements
1357822–1378644	20822 bp	5	4 (80%)	5 (100%)	hemolysin secretion/activation
2201422–2221045	19623 bp	22	18 (82%)	19 (86%)	mobile genetic elements
2233554–2244999	11445 bp	33	28 (85%)	31 (94%)	thiamin biosynthesis, peptidase
2324538–2330589	6051 bp	6	2 (33%)	6 (100%)	mangotoxin biosynthetic operon *mbo*
2710838–2721328	10490 bp	10	10 (100%)	10 (100%)	hypothetical proteins
3032080–3050678	18598 bp	14	13 (93%)	14 (100%)	hypothetical proteins
4684145–4696189	12044 bp	7	0	6 (86%)	cellulose synthase
5668482–5685647	17165 bp	21	21 (100%)	21 (100%)	hypothetical proteins

**Table 4:** Regions of *Pseudomonas syringae* pv. syringae B728a genome with low similarity to *P*. *syringae* pv. syringae UMAF0158.

Location (bp)	Length	No. of CDSs	No. of hypothetical	No. of CDSs not present in UMAF0158	Relevant features
102358–116873	14515 bp	12	9 (75%)	10 (83%)	hypothetical proteins/mobile genetic elements
838293–843261	4968 bp	6	1 (17%)	5 (83%)	hypothetical proteins/mobile genetic elements/T3 effector
1614863–1658265	43402 bp	46	37 (80%)	40 (87%)	T4 secretion components
1672580–1678069	5489 bp	8	6 (75%)	7 (88%)	hypothetical proteins/membrane transport
1692009–1713389	21380 bp	18	6 (33%)	14 (78%)	mobile genetic elements/secretion/pilus proteins
3182993–3199241	16248 bp	11	6 (55%)	10 (91%)	hypothetical proteins/putative virulence protein
3207940–3242247	34307 bp	23	12 (52%)	18 (78%)	streptomycin resistance
3368488–3409506	41018 bp	53	44 (83%)	44 (83%)	hypothetical proteins/phage-related proteins
4526331–4542603	16272 bp	11	7 (64%)	7 (64%)	hypothetical proteins/mobile genetic elements/T3 effector
5507043–5520779	13736 bp	14	6 (43%)	11 (79%)	hypothetical proteins/phage-related proteins/plasmid-related proteins/T3 effector

In addition, ten regions were identified in B728a with sizes ranging from 4968 to 43402 bp. Most of these regions contain mobile genetic elements. It is worth noting a region containing the streptomycin resistance transposon *Tn*5393 [12]. Two other regions are enriched in secretion components with one of them corresponding to a T4SS, which is addressed below.

### Secretion systems

**Fig 4:**
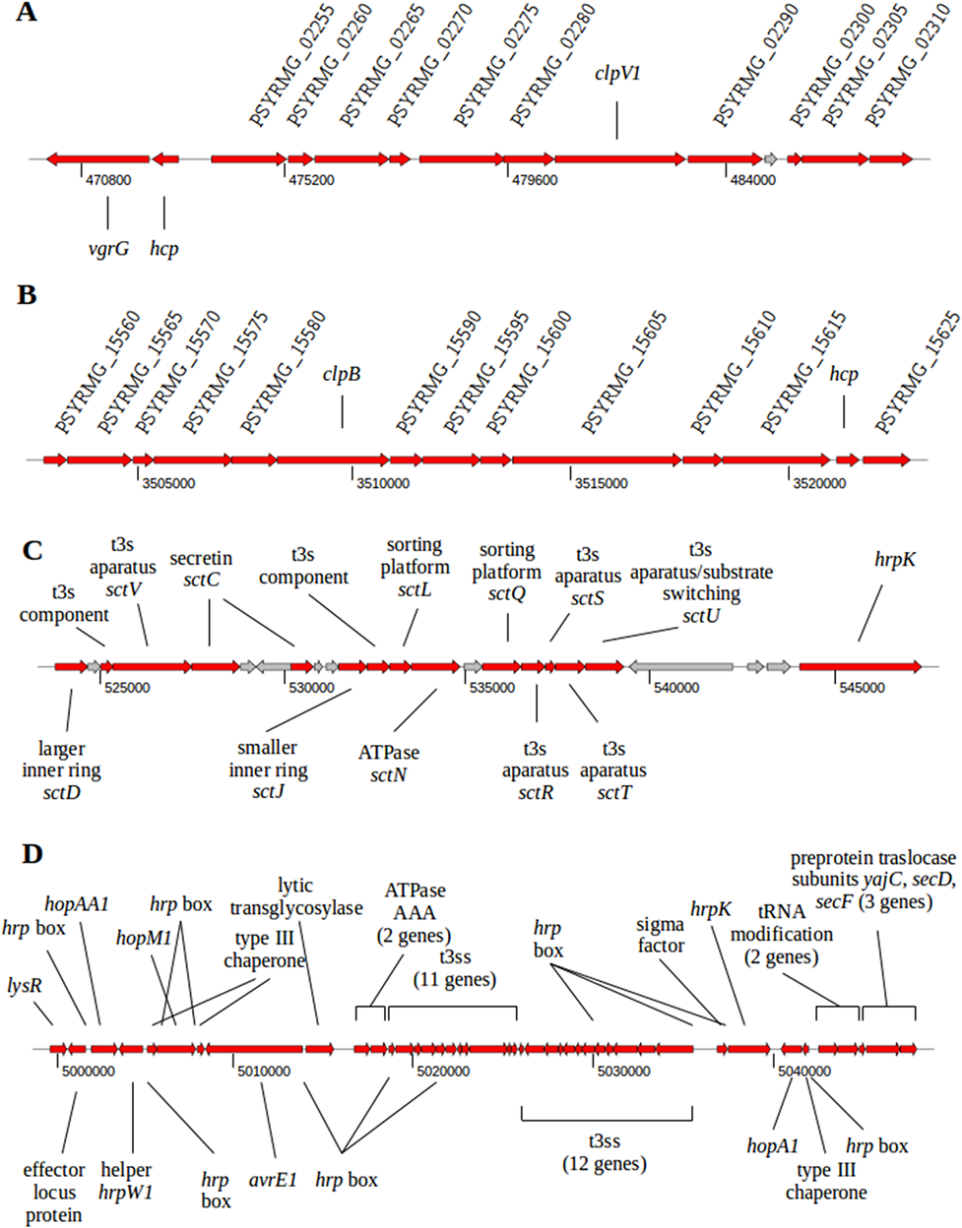
Genomic organization of putative *Pseudomonas syringae* pv. syringae UMAF0158 secretion systems involved in effector translocation. **A,** T6SS-1. **B,** T6SS-2. **C,** T3SS-1 (*hrc*-1). **D,** T3SS-2 (*rhc*). Genes presumably involved in secretion are shown in red. Components of the T6SS with no consensual name are labelled with their corresponding NCBI-annotated locus tags. Numbers below the arrows refer to bp positions in the chromosome.

The T6SS, which was first described in pathogenic bacteria such as *Vibrio cholerae*, *Pseudomonas aeruginosa* and *Burkholderia mallei* [44–46], has been widely identified in Gram-negative bacteria, including *P*. *syringae* [47]. This versatile secretion system has been proposed to promote symbiotic, commensal or mutualistic relationships between bacteria and eukaryotes and to intervene in cooperative or competitive interactions between different bacteria [48]. UMAF0158 contains two putative gene clusters that are associated with the T6SS, similarly to what it has been previously described for other strains of pvs. tomato, tabaci and oryzae, and in contrast with the genomes of B728a and 1448A, which carry only one T6SS [47]. Both clusters in UMAF0158 are composed of 14 genes (PSYRMG_02245 to PSYRMG_02310 and PSYRMG_15560 to PSYRMG_15625) and range from coordinates 470416 to 487611 (Fig 4A) and 3502757 to 3522874 (Fig 4B), respectively. The first cluster is highly conserved in DC3000 [49], while the second shows high similarity to that found in B728a [49].

The T4SS can be involved in the translocation of proteins and genetic material, thus contributing to genome plasticity and virulence of bacteria harboring them [50]. B728a contains a conjugative G-type T4SS [51] that was not found in UMAF0158. However, UMAF0158 encodes a putative P-type T4SS in its plasmid, which is presumably involved in DNA conjugation [42] (S4 Table).

The role of T3SSs in the virulence of pathogenic bacteria has been widely investigated [52,53]. Two T3SS clusters were observed in the UMAF0158 chromosome. A complete T3SS (here called T3SS-1) similar to the Hrp-1 family of T3SS [54] found in pathogenic *P*. *syringae* strains [55,56] was identified, ranging from coordinates 5000786 to 5047952 and consisting of 42 genes (PSYRMG_22290 to PSYRMG_22510) (Fig 4C). This was not unexpected as this strain has been shown to induce the hypersensitive response (HR) in tobacco plants [15], a process dependent on a functional T3SS in *P*. *syringae* [57]. This cluster showed high similarity to that found in B728a, whose role in virulence has been widely reported [58,59]. The second putative T3SS (here called T3SS-2), which ranges from coordinates 523773 to 547384 in the UMAF0158 chromosome and consists of 24 genes (PSYRMG_02470 to PSYRMG_02585) (Fig 4D), shows high similarity to the rhizobial-like T3SS Rhc of Rhizobiales family of T3SS [54,56]. Among these 24 genes are included the complete set of core components to form the minimal apparatus [60]. This cluster is also encoded in *P*. *syringae* pv. phaseolicola 1448A [13], *P*. *syringae* pv. tabaci 11528 [61], *P*. *syringae* pv. oryzae 1–6 [62], *P*. *syringae* pv. syringae 642 [63] and *P*. *savastanoi* NCPPB 3335 [24,64] among other strains of the *P*. *syringae* complex but not in *P*. *syringae* pv. tomato DC3000 and *P*. *syringae* pv. syringae B728a. In agreement with data reported for other *P*. *syringae* genomes, regulatory sequences typical of the HrpL regulon [65] were not found preceding the genes in this second T3SS cluster. As deduced from analysis of specific mutants in the canonical T3SS in other *P*. *syringae* strains also encoding this second T3SS [66,67], it has been suggested that this rhizobial-like T3SS Rhc is not essential for pathogenicity, but a possible role in plant surface colonization or interaction with insects has been postulated [63,68]. To address this hypothesis experimentally, we constructed a UMAF0158 T3SS-2 mutant and analyzed its ability to infect tomato plants (see below).

### Phenotypic analysis of a second T3SS (T3SS-2)

Defective simple mutants via deletion of the *hrpL* gene (UMAF0158Δ*hrpL*) and deletion of 2500 bp from the *rhc* cluster containing the *rhcJ*, *rhcL*, *rhcN* genes (UMAF0158Δ*rhc*), and the double mutant UMAF0158Δ*hrpL* + *rhc* and complemented mutant *UMAF0158Δ*hrpL + pLac-*hrpL* were constructed and assayed to search some evidence of T3SS-2 function. *In vitro* motility tests showed no differences, and swimming and swarming type movements did not appear to be affected by any of the mutations. Furthermore, experiments were performed *in planta* including a hypersensitivity response (HR) test in tobacco, surface adhesion on mango leaves, bacterial growth on the surface of tomato leaves and pathogenesis in tomato leaflets maintained *in vitro*. The wild type, T3SS-2 mutant (*UMAF0158Δ*rhc) and complemented T3SS-1 mutant (*UMAF0158Δ*hrpL+pLac::*hrpL*) demonstrated a typical HR reaction; however, the T3SS-1 mutant (*UMAF0158Δ*hrpL) and double mutant (*UMAF0158Δ*hrpL+*rhc*) did not induce a reaction in inoculated tobacco leaves as expected. In adhesion assays, the results obtained revealed an identical behavior for all strains assayed (S2 Fig). Similarly, the parental strain and simple and double mutants had no differences in survival assays on tomato leaflet surfaces. The pathogenicity assays on tomato leaflets showed no relevant results in either the incidence or severity of necrotic symptoms. Thus, the symptom incidence induced by the wild type and T3SS-2 and complemented T3SS-1 mutant strains demonstrated the highest levels. In contrast, the lowest symptoms for incidence were observed for the double mutant and single T3SS-1 mutant (S3 Fig). The severity of the symptoms demonstrated results similar to that for incidence. Finally, toxicity experiments in *Caenorhabditis elegans* and *Galleria mellonella* were performed, resulting in the complete absence of toxicity from the bacterial strains used, including the wild type and mutant strains. All of these experiments have not demonstrated a relevant role for the T3SS-2 of UMAF0158 in virulence, leaf colonization and toxicity in insects and worms as proposed by other authors for atypical *Pseudomonas* T3SSs [63,68].

### T3SS effectors

**Fig 5:**
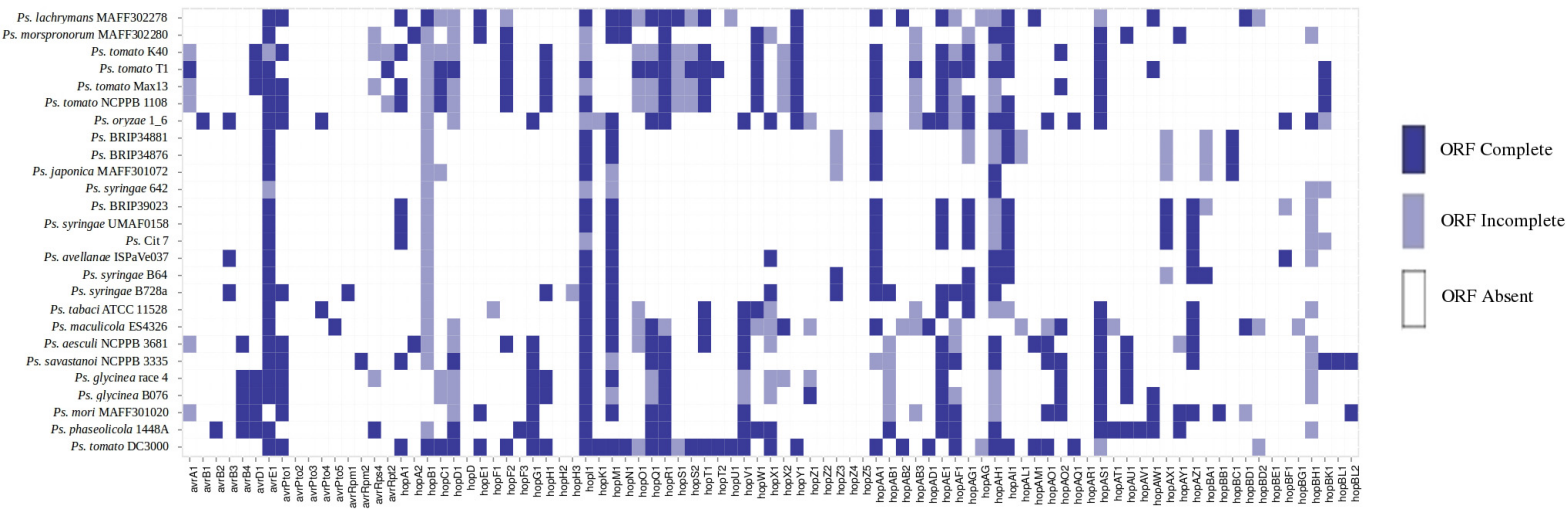
Presence of T3Es in 26 *Pseudomonas syringae* strains (see S1 Table). T3Es from the *P*. *syringae* Genome Resource (http://pseudomonas-syringae.org/) are listed across the bottom. Blue boxes indicate presence of complete ORFs within each genome; light blue boxes indicate that genes were found by similarity searches but they seem to be incomplete (see Materials and Methods section); white boxes indicate that no significant matches (*E*-value ≤ 1×10^−10^) were found. The alignments used to generate this figure have been included as supporting information in S3 File and S2 Table, which contains detailed information on these analyses.

**Fig 6:**
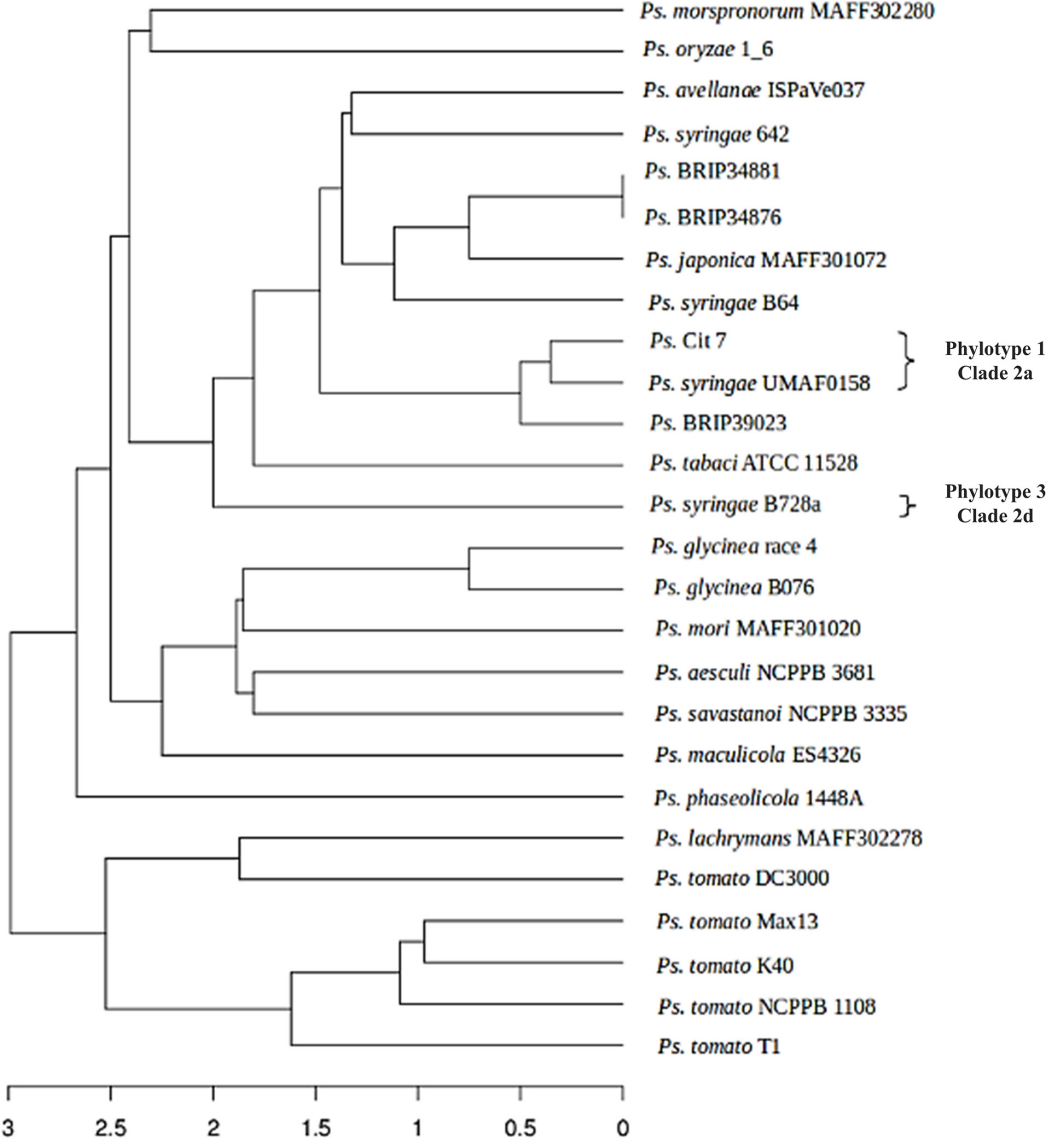
Dendrogram analysis of *Pseudomonas syringae* pv. syringae UMAF0158 and 25 selected strains of the *P*. *syringae* complex (see S1 Table) based on the presence of T3Es. A matrix was created based on the presence/absence of T3E proteins (see supporting information S9 Table). Then, the corresponding distance matrix was inferred, and the R package APE was used to generate the tree (see Materials and Methods section). The scale shows the joining distance between each join point (strains) in the matrix. Some strains were labelled with the corresponding phylotype of pv. syringae [21, 22] and clade of phylogroup 2 of *P*. *syringae* complex [6].

The *in silico* proteomes of UMAF0158 and the 25 selected *P*. *syringae* strains listed in S1 Table were screened for known *P*. *syringae* T3Es. Based on the presence or absence (*E*-value ≤ 1x10^-10^) of the 89 effector proteins currently described in http://pseudomonas-syringae.org/, a matrix was constructed by evaluating whether each T3E was found as a complete ORF, an incomplete ORF, or not present (Fig 5). S2 Table contains detailed information on these analyses. By selecting T3Es with complete and incomplete ORFs, a matrix was built based on Fig 5 data and used to generate a dendrogram shown in Fig 6. Note that we accounted for incomplete ORFs, since truncated effectors can still be effective *in planta* [64]. The results showed that UMAF0158 encodes 11 putative T3Es, including 7 which are also encoded in the B728a genome. Notably, and in agreement with the phylogeny showed in Fig 2, Cit 7 was the closest strain to UMAF0158, both containing the same T3Es repertoire, including *hopI1* which is disrupted in Cit7.

### Virulence Factors

The UMAF0158 genome was analyzed to detect known genes potentially implicated in virulence. We interpreted virulence to include factors such as siderophores, adhesins, phytotoxins, phytohormones, detoxifying compounds, plant cell wall degrading enzymes (PCWDEs) and exopolysaccharides (EPSs). In total, we identified 107 putative orthologs involved in the production of any of the above virulence factors (listed in S7 Table and summarized in Table 5), which are addressed below, most of them also present in B728a.

**Table 5:** Summary of putative virulence-associated genes in *Pseudomonas syringae* pv. syringae UMAF0158.

Virulence Factor	Products
Toxins	Syringomycin, syringopeptin, mangotoxin, phaseolotoxin, syringolin A
PCWDEs	Pectin lyase, xylanase, cellulase, lipoyl synthase
Siderophores	Achromobactin, pyoverdine
Adhesion proteins	Cellulose synthase, filamentous haemagglutinin, fimbrial proteins, adhesion proteins *attC/attG*
Detoxifying compounds	Copper oxidase, catalase/hydroperoxidase, proline iminopeptidase, ferritin, cytochrome C oxidase
Phytohormones	Auxin
EPSs	Alginate, *gpsX*

#### Siderophores

Phytopathogenic bacteria synthesize and secrete a number of low molecular weight iron-chelating compounds called siderophores, which allow them to grow in iron-limited host environments [69,70]. UMAF0158 encodes genes orthologous to those required for the synthesis of pyoverdin and achromobactin, which have been widely described in other *Pseudomonas* [12,71,72]. These two siderophores have also been proposed to increase the epiphytic fitness of *P*. *syringae* [73].

#### Adhesins and exopolysaccharides (EPSs)

Once a target host is reached, bacteria activate machinery for adhesion to plant tissues, which is thought to be necessary for the pathogenesis of many strains [74,75]. Attachment factors previously identified in the genome of *P*. *syringae* strains include type IV pili, alginate, non-alginate capsular polysaccharide, EPSs, and filamentous hemagglutinin [13]. Several genes predicting adhesins have been found in the UMAF0158 genome, including a filamentous hemagglutinin (PSYRMG_05745) and several fimbrial proteins, with the latter being clustered together with a number of pilus assembly proteins (see additional file 9). Another gene predicting an adhesin is PSYRMG_14835, which resembles the gene encoding XadM, whose role in attachment and the formation of biofilms has been previously reported in *Xanthomonas oryzae* [76]. Interestingly, UMAF0158 contains two genes showing high similarity with the *attC* and *attG* genes in *Agrobacterium* (PSYRMG_02675 and PSYRMG_09205, respectively), whose mutation leads to virulence and lack of attachment on tomato, carrot, and *Bryophyllum daigremontiana* [77].

Synthesis and secretion of EPSs is a common mechanism used by phytopathogenic bacteria, particularly in *P*. *syringae* strains [7]. They contribute to virulence by helping in attachment to host tissues and protecting bacterial cells from external stress [78]. All of the genes required for alginate biosynthesis [79] are present in the UMAF0158 gene cluster (PSYRMG_21640–21695). Notably, an ortholog of *gpsX*, a gene that encodes a glycosyltransferase that is involved in EPS production and is essential for the full virulence of *Xanthomonas citri* [80], which was also identified (PSYRMG_21000).

#### Phytotoxins

The production of small phytotoxic compounds by *P*. *syringae* pv. syringae is a well-known virulence mechanism that contributes to plant disease [17,81]. UMAF0158 and B728a contain orthologs of genes participating in the synthesis of syringopeptin and syringomycin. These two toxins induce necrosis in plant tissues and have been shown to be the major virulence determinants of *P*. *syringae* pv. syringae [16,82]. The two clusters encoding these toxins form a larger cluster (PSYRMG_03860–03910), which is consistent with data previously reported [12,82]. The production of syringomycin by UMAF0158 has been experimentally validated in previous studies by our group [17].

Syringolins are another family of phytotoxins synthesized by a number of *P*. *syringae* pv. syringae [83]. UMAF0158 has a gene cluster resembling that of the production of syringolin A (PSYRMG_24250–24275), a toxin that has been shown to counteract stomatal innate immunity in beans and *Arabidopsis* [84].

Phaseolotoxin and coronatine are two chlorosis-inducing toxins that also represent major virulence factors for some *P*. *syringae* isolates [85,86]. UMAF0158 lacks orthologs for most of the genes involved in the production of coronatine; however, analysis of the 23 genes required for the synthesis of phaseolotoxin [86] showed that orthologs of 17 of these genes are included in its genome. Given that no inhibition halos were observed in the bioassay for toxin detection when ornithine was added [17,18] and no chlorosis was detected among the symptoms of UMAF0158 infection, it is likely that the lack of the other six genes prevent the synthesis of phaseolotoxin by this strain.

The production of mangotoxin by UMAF0158 and its contribution to the virulence of this strain has been widely described [17–19]. Two operons are involved in the synthesis of this toxin, including *mgo* (PSYRMG_15820–15835) and *mbo* (PSYRMG_10110–10135) [18–21], and the latter is absent in B728a as previously described. Unlike other toxins, mangotoxin is thought to be associated with host specificity because it is typically synthesized by strains of phylotypes 1 and 2 of pv. syringae, which were mainly isolated from mango trees and other woody crops [22].

It is worth noting that the production of at least two phytotoxins has been experimentally validated in UMAF0158 (i.e., syringomicin and mangotoxin) [17,18]. Whether this strain is capable of producing the rest of the phytotoxins mentioned above is a question that requires further investigation.

#### Phytohormones

Bacterial-produced phytohormones are typically transported to the plant cell to regulate plant biological processes, providing a beneficial context for the pathogen [87]. Such is the case of auxin, which is predominantly represented by indole-3-acetic acid (IAA), a key plant growth regulator that is also involved in plant-bacteria interactions. The downregulation of this hormone in plants has been shown to restrict *P*. *syringae* growth in *Arabidopsis* [88], suggesting that bacteria may have evolved the production of auxin to overcome this plant response. Accordingly, auxin production has been demonstrated to promote susceptibility to *P*. *syringae* [89]. Although the two genes involved in the biosynthesis of IAA, *iaaH* and *iaaM*, are present in UMAF0158 (PSYRMG_14175 and PSYRMG_11680, respectively), IAA production was not detected in culture supernatants of this strain using a colorimetric assay [90]. Under the same conditions, IAA production was neither detected for *P*. *syringae* DC3000, which has been reported to produce low levels of IAA [11]. Thus, production of IAA by UMAF0158 in comparison to other *P*. *syringae* strains remains to be elucidated using more sensitive analytical methods and different culture conditions.

#### Detoxifying compounds

As a response to bacterial infections, plants synthesize reactive oxygen species (ROS), such as hydrogen peroxide (H_2_O_2_), superoxide (O_2_^-^) and hydroxyl radical (OH). These molecules have a toxic effect on invading bacteria [91], which have evolved mechanisms to counterattack by detoxification. DC3000 typically makes use of the catalases KatB and KatE together with the catalase-peroxidase KatG to detoxify plant-produced H_2_O_2_. Interestingly, orthologs of these three gene products are present in the UMAF1058 genome (see additional file 9). Moreover, an ortholog of the gene that encodes Dps (PSYRMG_23380), a ferritin-like protein that has been shown to protect plant-associated bacteria against oxidative stress [92], has also been identified. Other genes possibly implicated in detoxification found in UMAF0158 correspond to a cluster presumably encoding a cbb(3)-type cytochrome C oxidase (PSYRMG_07490–07505) and a proline iminopeptidase (PSYRMG_18265). The latter has been reported to be required for pathogenicity of *X*. *campestris* [93] and to have dealanylating activity toward ascomycin, an antibiotic produced by *Streptomyces* that inhibits protein synthesis [94]. Genes involved in copper resistance have also been identified, including a cluster containing *copA* and *copB* (PSYRMG_23630 and PSYRMG_23625, respectively), and a locus with high similarity to the *cueAR* system in *P*. *putida* [95]. This system consists of a copper-transporting ATPase transmembrane protein and its transcriptional regulator (PSYRMG_19635 and PSYRMG_19630, respectively). Additionally, the *copABCD* operon described in other *P*. *syringae* [96] is absent in the UMAF0158 chromosome, and also any copper resistant genes are present in the UMAF0158 plasmid, in agreement with the copper sensitivity of this strain [97].

#### Plant cell wall degrading enzymes (PCWDEs)

Some phytopathogenic bacteria need to overcome the plant cell wall in the process of accessing the host cytoplasm. Therefore, many plant pathogens harbor a collection of genes encoding PCWDEs, which are considered important virulence determinants [98]. The UMAF1058 genome contains genes predicting a cellulase (PSYRMG_06950), a lipoyl synthase (PSYRMG_12655), a xylanase (PSYRMG_13355) and a pectin lyase (PSYRMG_10750), which are also detected in B728a [12] and other *P*. *syringae* strains [13,99]. Although there is no experimental evidence in this case, it could be assumed that the above predicted enzymes are likely exported by means of a type II secretion system (T2SS) putatively encoded in the genome of UMAF0158.

## Conclusions

Summarizing, bioinformatic analysis of the complete genome of *P*. *syringae* pv. syringae UMAF0158, a pathogen of mango trees, revealed a high degree of conservation with other pseudomonads belonging to the *P*. *syringae* complex, including the model strain *P*. *syringae* pv. syringae B728a. However, the resulted phylogeny clustered UMAF0158 with *P*. *syringae* Cit 7 and more separately from B728a. Indeed, our data revealed a number of genetic factors that could be involved in the differential pathogenic and epiphytic lifestyle of UMAF0158, in comparison with the model strain B728a. The mangotoxin biosynthetic operon *mbo* is included among these factors, which role in the pathogenicity of UMAF0158 has been previously reported [19,41]. Moreover, UMAF0158 harbors an operon involved in cellulose production and encodes additional T3SS and T6SS, as well as displays a particular T3Es repertoire. However, an UMAF0158 mutant affected in this rhizobial-like second T3SS (T3SS-2) showed identical virulence, leaf colonization ability and toxicity on insects or worms than the wild-type strain. Additionally, the conjugative plasmid pPSS158 harbors *rulAB* genes involved in UV resistance and epiphytic fitness [42]. This work provides the basis for further analysis on the specific mechanisms that enable this strain to infect mango trees and for the functional analysis of the factors governing host specificity in pv. syringae strains from different phylotypes.

## Supporting Information

S1 Fig Genomic representation of the secretion-associated features of *Pseudomonas syringae* pv. syringae UMAF0158. From the outside in, the outermost circle (black) shows the scale line; circles 2 represents T3SS (blue) and T6SS (green); circle 3 displays putative T3 effectors; circle 4 depicts predicted *hrp* boxes. Only secretion systems associated with effector translocation were considered.
(PDF)

S2 Fig Bacterial cells counts recovered during adhesion experiments on mango leaves. Drops of bacterial suspension were deposited on mango leaves, after 30 min were softly washed and the adhered cell were recovered and counted. In this experiment were assayed *Pseudomonas syringae* pv. syringae UMAF0158 as wild type, and their defective simple mutants by deletion of *hrpL* gene (Δ*hrpL*) and deletion of 2500 bp of *rhc* cluster corresponding to *rhcJ*, *rhcL*, *rhcN* genes (Δ*rhc*), and a double mutant (Δ*hrpL* + *rhc*). The experimental data used to construct this figure are summarized as a datasheet in S8 Table.
(PDF)

S3 Fig Analysis of the two T3SS cluster as putative virulence factor of *Pseudomonas syringae* pv. syringae UMAF0158. The wild type *P*. *syringae* pv. syringae UMAF0158 and their defective simple mutants by deletion of *hrpL* gene (Δ*hrpL*) and deletion of 2500 bp of *rhc* cluster corresponding to *rhcJ*, *rhcL*, *rhcN* genes (Δ*rhc*), the double mutant (Δ*hrpL* + *rhc*) and the complemented mutant Δ*hrpL* + pLac-*hrpL* were inoculated into tomato leaflets by piercing and maintained in vitro ten days at 22°C and 16 h of photoperiod. Development of necrotic symptoms in tomato leaflets inoculated with the assayed strains were determined as the Incidence level of necrotic symptoms, it is represented as accumulative number of inoculated points developing necrotic area between 0.2 and 0.5 cm (cat. 2) and equal or higher than 5 mm in diameter (cat 3). The symptoms were monitoring and counted at different days from 0 to 10 for the total of the inoculated points with each strain. The ANOVA statistical analysis of severity was performed using data of tenth day. Asterisk mark significant differences regarding to wild type, double asterisk mark statistical differences regarding wild type and Δ*hrpL* mutant.
(PDF)

S1 File Complete Map Report of UMAF0158 Genome Sequencing Project. (PDF)

S2 File Alignment of the concatenated sequences corresponding to five house-keeping genes (*gapA*, *gltA*, *recA*, *rpoA* and *rpoB*) used to generate the phylogeny for the strains in Fig 2. Alignment of the concatenated protein sequences were used in Fig 2A (SX file) and the concatenated nucleotide sequences to generate the phylogeny of Fig 2B (SY file).
(ZIP)

S3 File Alignments of the analysed T3Es used to construct Figs 5 and 6. The information about T3Es repertoires found in 26 *Pseudomonas syringae* strains used in this study are provided in S2 Table.
(ZIP)

S1 Table Accession numbers and references of genome sequences corresponding to 26 *Pseudomonas* strains used in this study. (DOCX)

S2 Table T3Es repertoires found in 26 *Pseudomonas syringae* strains. Columns provide information on BLASTp alignments between T3Es from http://pseudomonas-syringae.org/ and strains gene products, such as *E*-value, fraction and number of identical positions, alignment length, coordinates (start-end) for the query effector and subject gene product in the alignment and number of gaps. Other relevant information is also provided, such as lengths of both the effector and the gene product, the rate between effector length and alignment length and whether the searched effectors are considered complete.
(XLSX)

S3 Table Primers used in the construction of TTSS mutants of *Pseudomonas syringae* pv. syringae UMAF0158. (DOC)

S4 Table Predicted ORF in *Pseudomonas syringae* pv. syringae plasmid (pPSS158, Gene Bank accession number CP005971). ORFs were first predicted and annotated by the NCBI Prokaryotic Genome Annotation Pipeline. Then, annotation was manually curated. ORFs highlighted in grey correspond to T4SS components (VirB, VirD), replication protein A and ultraviolet light resistance proteins A and B.
(DOC)

S5 Table Genes corresponding to regions of *Pseudomonas syringae* pv. syringae UMAF0158 genome with low similarity to *P*. *syringae* pv. syringae B728a (summarized in Table 3). Gray shading indicates genes which are present in B728a (*E*-value < 1e^-10^).
(DOCX)

S6 Table Genes corresponding to regions of *Pseudomonas syringae* pv. syringae B728a genome with low similarity to *P*. *syringae* pv. syringae UMAF0158 (summarized in Table 4). Gray shading indicates genes which are present in UMAF0158 (*E*-value < 1e^-10^).
(DOCX)

S7 Table Relevant virulence factors found in *P*. *syringae* pv. syringae UMAF0158 genome. Each row corresponds to an ORF. Columns provide information on locus tag, position (bp), strand, length (aa), protein product, type of virulence factor assigned and name of such a factor.
(XLSX)

S8 Table Datasheet including the original data of the adhesion on mango leaves experiments. The data for each replicate and experiment, and the calculated averages and standard deviations used to construct the supporting S2 Fig are summarized in this table.
(XLS)

S9 Table Matrix based on the presence/absence of putative T3Es in 26 *Pseudomonas syringae* strains. Complete, incomplete and not present ORFs are assigned values of 1, 0.5 and 0, respectively. This matrix was used to construct Fig 6.
(XLSX)
