# Supplementary material for: Bioinformatics Analysis of the Complete Genome Sequence of the Mango Tree Pathogen Pseudomonas syringae pv. syringae UMAF0158 Reveals Traits Relevant to Virulence and Epiphytic Lifestyle
Source: PLoS One. 2015 Aug 27;10(8):e0136101. doi: 10.1371/journal.pone.0136101 (PMC4551802; doi:10.1371/journal.pone.0136101)
Supplement: S1 Fig — From the outside in, the outermost circle (black) shows the scale line; circles 2 represents T3SS (blue) and T6SS (green); circle 3 displays putative T3 effectors; circle 4 depicts predicted hrp boxes. Only secretion systems associated with effector translocation were considered. (PDF) [file pone.0136101.s001.pdf]

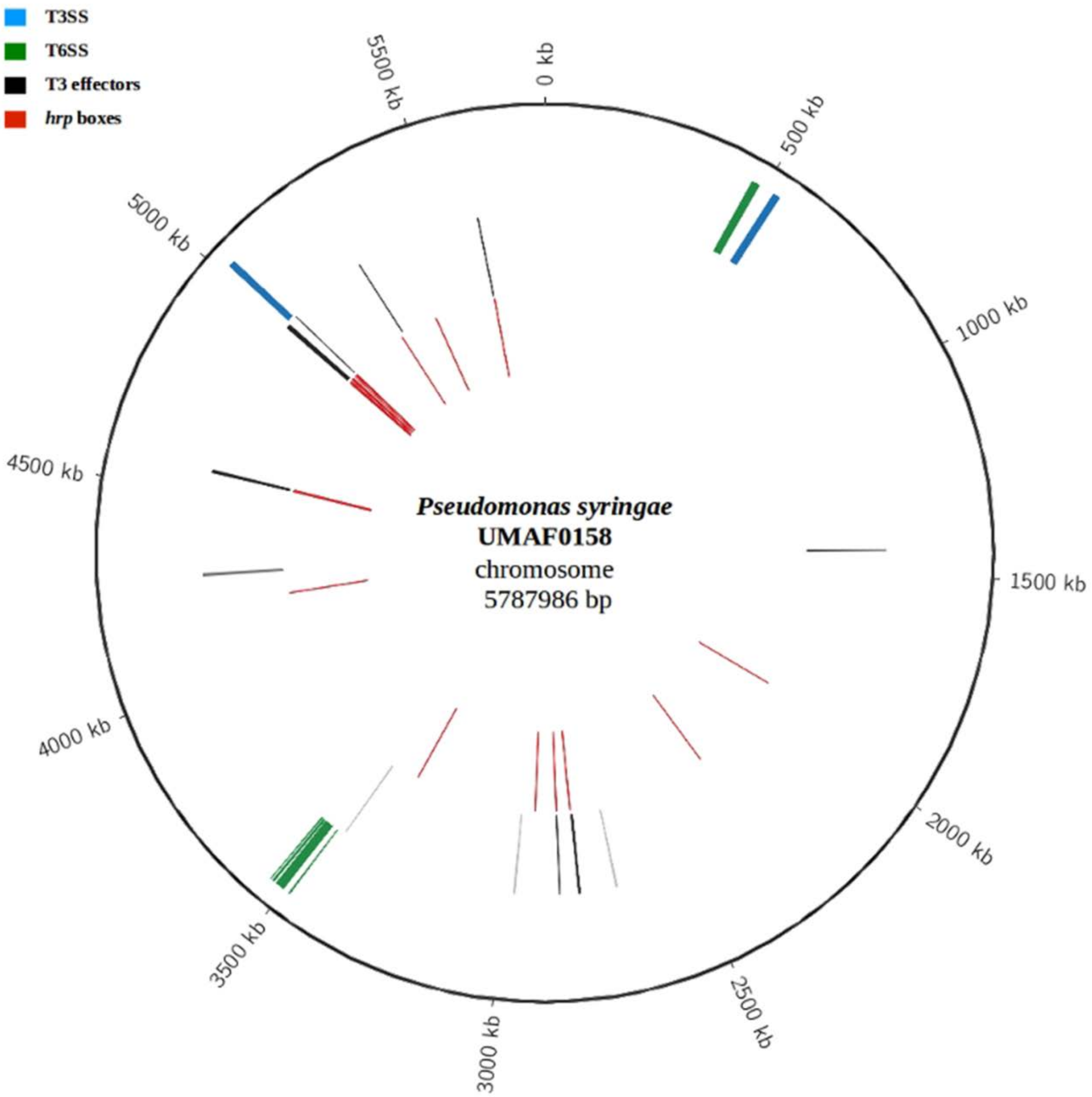

Figure S1 (additional file 5). Genomic representation of the secretion-associated features of *Pseudomonas syringae* pv. syringae UMAF0158. From the outside in, the outermost circle (black) shows the scale line; circles 2 represents T3SS (blue) and T6SS (green); circle 3 displays putative T3 effectors; circle 4 depicts predicted *hrp* boxes. Only secretion systems associated with effector translocation were considered.
