# Supplementary material for: Bioinformatics Analysis of the Complete Genome Sequence of the Mango Tree Pathogen Pseudomonas syringae pv. syringae UMAF0158 Reveals Traits Relevant to Virulence and Epiphytic Lifestyle
Source: PLoS One. 2015 Aug 27;10(8):e0136101. doi: 10.1371/journal.pone.0136101 (PMC4551802; doi:10.1371/journal.pone.0136101)
Supplement: S2 Fig — Drops of bacterial suspension were deposited on mango leaves, after 30 min were softly washed and the adhered cell were recovered and counted. In this experiment were assayed Pseudomonas syringae pv. syringae UMAF0158 as wild type, and their defective simple mutants by deletion of hrpL gene (ΔhrpL) and deletion of 2500 bp of rhc cluster corresponding to rhcJ, rhcL, rhcN genes (Δrhc), and a double mutant (ΔhrpL + rhc). The experimental data used to construct this figure are summarized as a datasheet in S8 Table. (PDF) [file pone.0136101.s002.pdf]

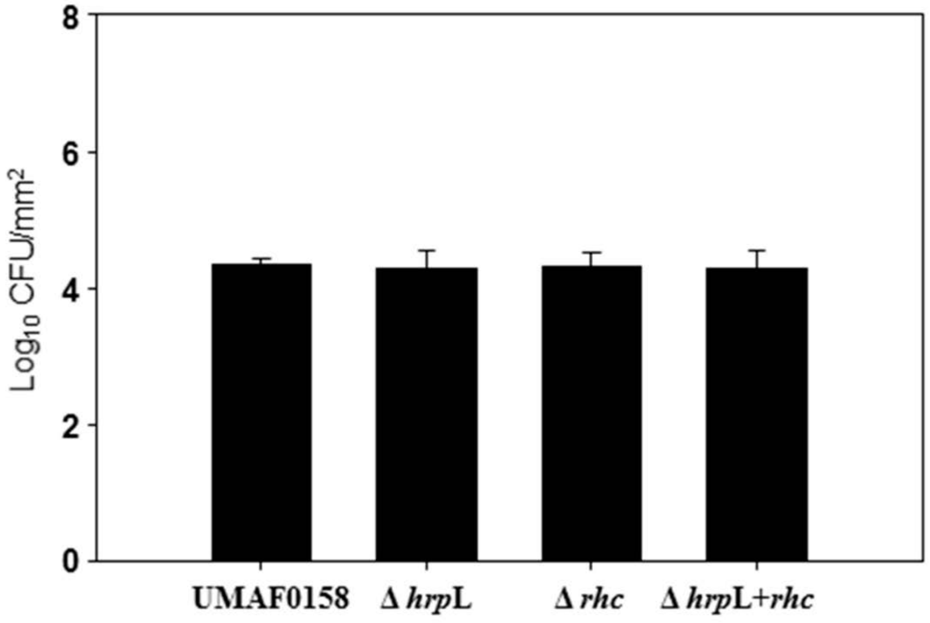

Figure S2 (additional file 6). Bacterial cells counts recovered during adhesion experiments on mango leaves. Drops of bacterial suspension were deposited on mango leaves, after 30 min were softly washed and the adhered cell were recovered and counted. In this experiment were assayed *Pseudomonas syringae* pv. syringae UMAF0158 as wild type, and their defective simple mutants by deletion of *hrpL* gene (Δ*hrpL*) and deletion of 2500 bp of *rhc* cluster corresponding to *rhcJ*, *rhcL*, *rhcN* genes (Δ*rhc*), and a double mutant (Δ*hrpL* + *rhc*).
