# Supplementary material for: Bioinformatics Analysis of the Complete Genome Sequence of the Mango Tree Pathogen Pseudomonas syringae pv. syringae UMAF0158 Reveals Traits Relevant to Virulence and Epiphytic Lifestyle
Source: PLoS One. 2015 Aug 27;10(8):e0136101. doi: 10.1371/journal.pone.0136101 (PMC4551802; doi:10.1371/journal.pone.0136101)
Supplement: S3 Fig — The wild type P. syringae pv. syringae UMAF0158 and their defective simple mutants by deletion of hrpL gene (ΔhrpL) and deletion of 2500 bp of rhc cluster corresponding to rhcJ, rhcL, rhcN genes (Δrhc), the double mutant (ΔhrpL + rhc) and the complemented mutant ΔhrpL + pLac-hrpL were inoculated into tomato leaflets by piercing and maintained in vitro ten days at 22°C and 16 h of photoperiod. Development of necrotic symptoms in tomato leaflets inoculated with the assayed strains were determined as the Incidence level of necrotic symptoms, it is represented as accumulative number of inoculated points developing necrotic area between 0.2 and 0.5 cm (cat. 2) and equal or higher than 5 mm in diameter (cat 3). The symptoms were monitoring and counted at different days from 0 to 10 for the total of the inoculated points with each strain. The ANOVA statistical analysis of severity was performed using data of tenth day. Asterisk mark significant differences regarding to wild type, double asterisk mark statistical differences regarding wild type and ΔhrpL mutant. (PDF) [file pone.0136101.s003.pdf]

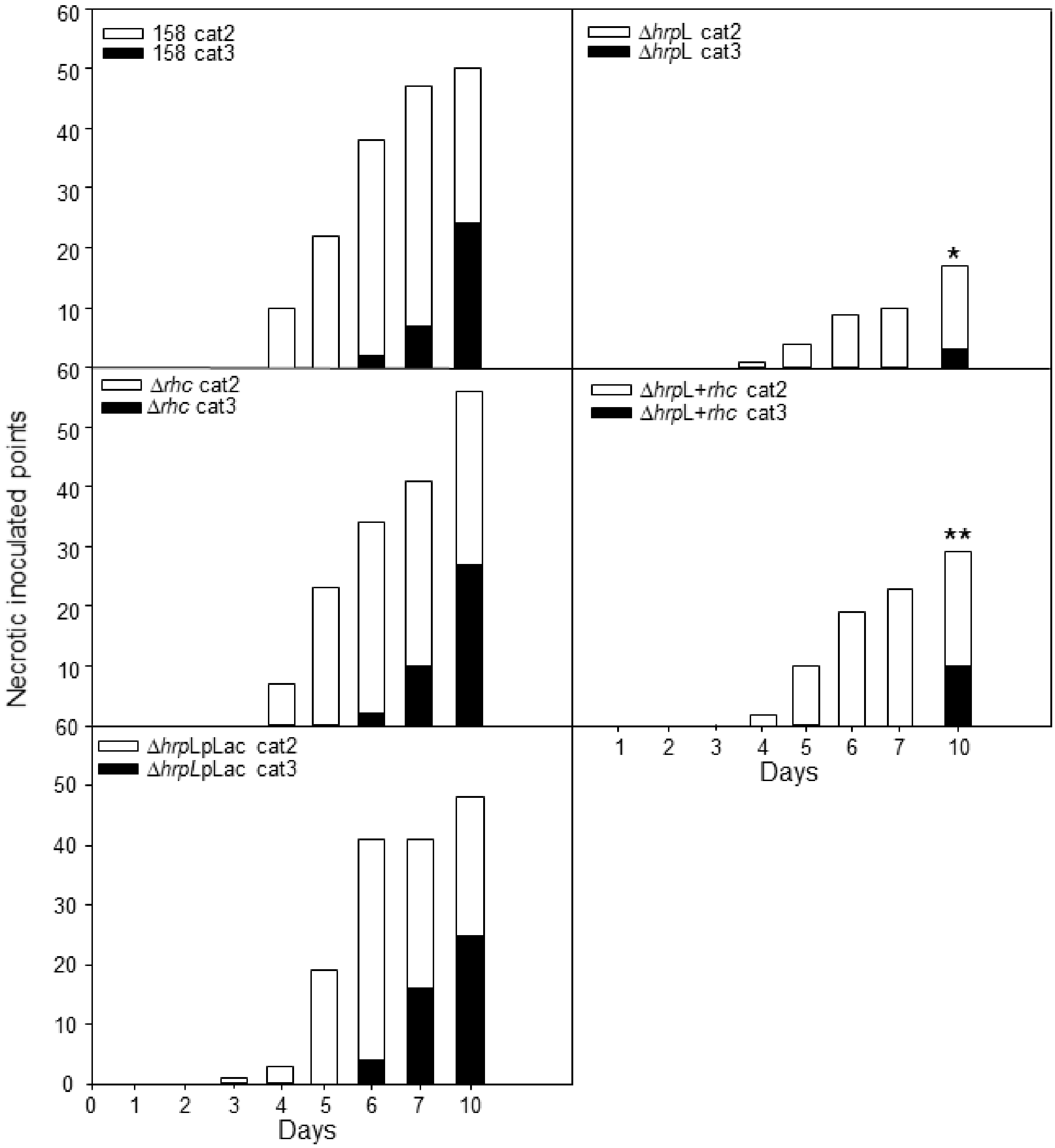

Figure S3 (additional file 7). Analysis of the two T3SS cluster as putative virulence factor of *Pseudomonas syringae* pv. syringae UMAF0158. The wild type *P. syringae* pv. syringae UMAF0158 and their defective simple mutants by deletion of *hrpL* gene (Δ*hrpL*) and deletion of 2500 bp of *rhc* cluster corresponding to *rhcJ*, *rhcL*, *rhcN* genes (Δ*rhc*), the double mutant (Δ*hrpL* + *rhc*) and the complemented mutant Δ*hrpL* + pLac-*hrpL* were inoculated into tomato leaflets by piercing and maintained in vitro ten days at 22ºC and 16 h of photoperiod. Development of necrotic symptoms in tomato leaflets inoculated with the assayed strains were determined as the Incidence level of necrotic symptoms, it is represented as accumulative number of inoculated points developing necrotic area between 0.2 and 0.5 cm (cat. 2) and equal or higher than 5 mm in diameter (cat 3). The symptoms were monitoring and counted at different days from 0 to 10 for the total of the inoculated points with each strain. The ANOVA statistical analysis of severity was performed using data of tenth day. Asterisk mark significant differences regarding to wild type, double asterisk mark statistical differences regarding wild type and Δ*hrpL* mutant.
