# Supplementary material for: Bioinformatics Analysis of the Complete Genome Sequence of the Mango Tree Pathogen Pseudomonas syringae pv. syringae UMAF0158 Reveals Traits Relevant to Virulence and Epiphytic Lifestyle
Source: PLoS One. 2015 Aug 27;10(8):e0136101. doi: 10.1371/journal.pone.0136101 (PMC4551802; doi:10.1371/journal.pone.0136101)
Supplement: S1 File — (PDF) [file pone.0136101.s004.pdf]

华大基因 BGI | Premier Scientific Partner

Complete Map Report of Bacteria Genome Sequencing Project

Bioinformatics Center

Version 1.0

May 17th, 2012

CONTENT

1 CONVETIONS 1

2 PIPELINE DESCRIPTIONS 2

2.1 Bacteria Complete Map Analysis Pipeline Description 2

2.2 Bioinformatics Analysis Pipeline Description 2

3 SEQUENCING DATA STATISTIC 4

3.1 Solexa Sequencing Data 4

3.1.1 Method 4

3.1.2 Result 5

4 GENOME SURVEY RESULT 8

4.1 Assembly Result 8

4.2 Survey Assessment Result 9

5 COMPLETE MAP ASSEMBLY ANALYSIS 10

5.1 Method 10

5.2 Assembly Result before PCR 12

6 PCR FILL GAP AND VERIFICATION 13

6.1 Method 13

6.2 Result 14

7 ASSEMBLY QUALITY CONTROL 16

7.1 Method 16

7.2 Result 18

8 RESULT FEEDBACK 19

8.1 Assembly Result Statistics 19

8.2 Repeat Sequence Statistics 19

10 DATA DOWNLOADING 21

11 CONTACT US 22

1 CONVETIONS

Table 1.1 Notational convention used in this document

Notation	Description
Italic	A Latin name for species, for example：”*Pseudomonas aeruginosa PAO1*”.
Italic	A web site. For example: “http://soap.genomics.org.cn/soapdenovo.html; version: 1.05 ”.
**Bold-normal**	Title. For example: ”**1 CONVENTIONS**”.
Normal	Text. For example: ”Sequencing data will be mapped to reference genome”.

2 PIPELINE DESCRIPTIONS

2.1 Bacteria Complete Map Analysis Pipeline Description

Bacteria complete map product contains two steps: genome detection, complete map assembly. The detailed analysis pipeline is shown below.

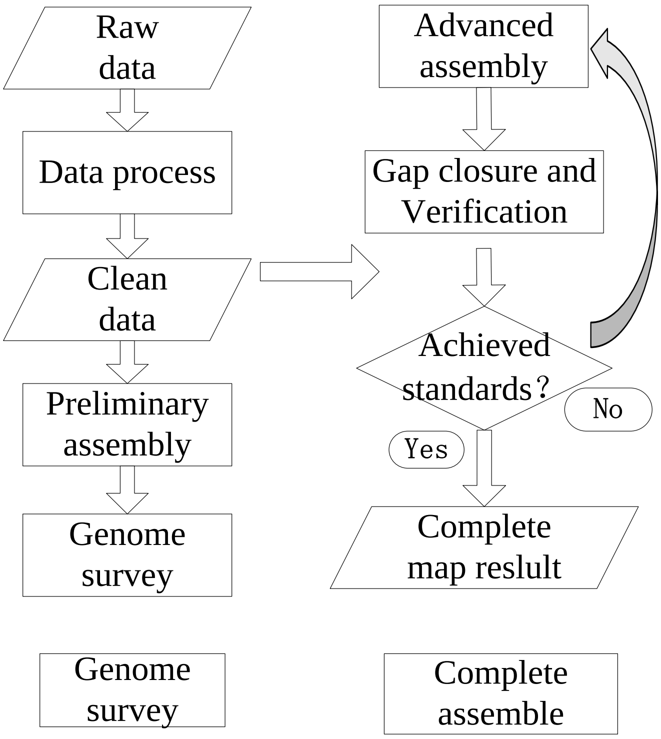

Figure 2.1 Bacteria complete map analysis pipeline

(1) Genome survey. The aim of this step is to obtain target strain genome information and sample status (genome size, GC content, repeat sequence, plasmid, heterogeneous sequence etc.) quickly through bioinformatics methods.

(2) Complete map assembly. The aim of this step is to assemble target strain genome sequence to one contig. Considering target strain genome complexity, multiple assembly softwares and gap closure methods will be applied to assemble sequence and optimize the assembly result. Unreliable region will be verified by two times PCR.

2.2 Bioinformatics Analysis Pipeline Description

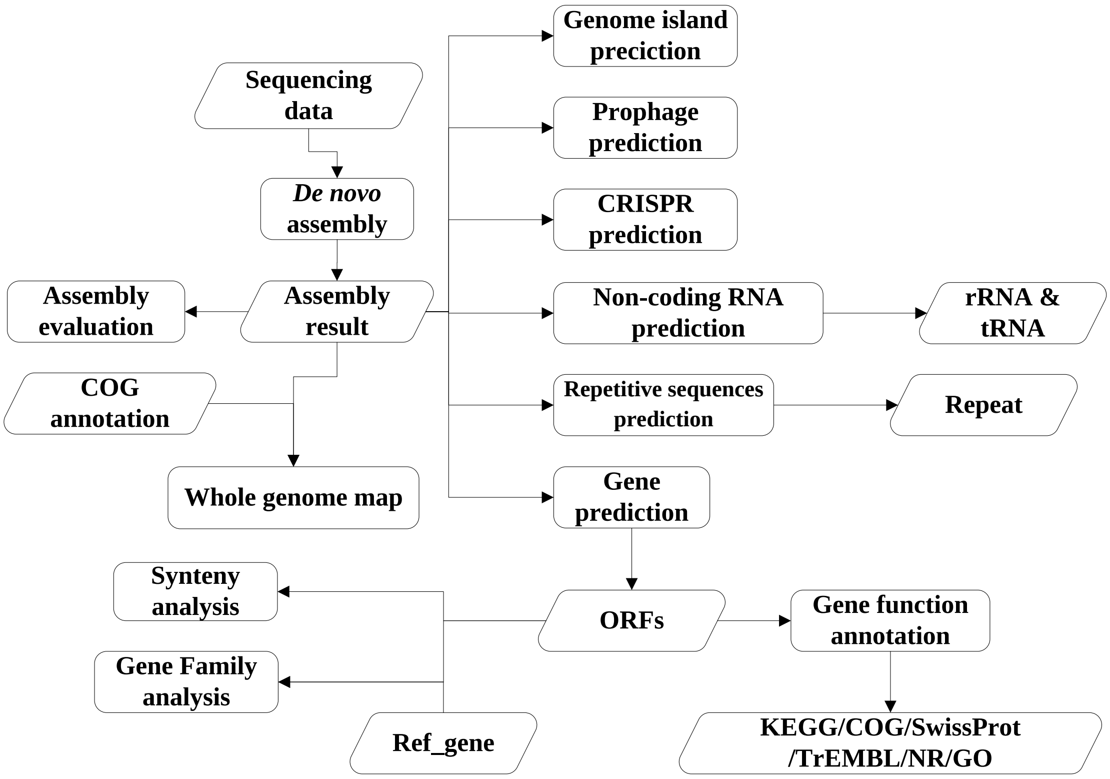

Figure 2.2 Pipeline of advanced bioinformatics analysis. (1) Data filtering. Filter the rawdata; (2) Assembly. Use SOAPdenovo (version 1.05) which is a short sequence assembly software to assemble the reads after filtering; (3) Evaluation of the assembly results. If there is a near-source reference sequence,we can obtain the evaluation of gene region of the genome and coverage; (4) Non-codingRNA prediction. We find the rRNA by comparison with the reference, or Use rRNAmmer software to predict rRNA; tRNA region and its secondary structure are predicted by tRNAscan software; sRNA is predicted by Rfam software; (5) Repetitive sequences analysis. RepeatMasker software (using Repbase database) and RepeatProteinMasker software (using the RepeatMasker library that comes with transposon protein) are two methods to predict the transposon; and tandem repeats are predicted by TRF (Tandem Repeat Finder) software; (6) Gene prediction. Gene sequences are obtained from assembly result using Glimmer3.0 software; (7) Gene functional annotation. The corresponding functional annotation information can be obtained by comparing the gene sequences with the databases; (8) Whole genome map (circle). A GC diagram is plotted according to the genome sequence, GC skew analysis is calculated by (G-C)/(G+C) method, and the distribution of COG annotated gene in the genome is plotted based on the annotation result and the gene locus; (9) Synteny analysis. The protein orDNA aggregates of

target bacterium is aligned with the protein or DNA aggregates of reference bacterium ,Two proteins or DNA with best hit for two alignments are marked in the coordinate diagram according to its position information after the same proportion of size reduction; (10) Genome island preciction. We carry out BLAT（Standalone BLAT v. 34）alignment between target genome and the genome island database set by the genome island sequence predicted by SIGI-HMM software, the possible GIs will be predicted; (11) Prophage prediction. We perform BLAT comparison（Standalone BLAT v. 34） between sequenced genome and prophage database which thhe prophage sequences are from Prohinder software and ACLAME database, the possible prophage will be predicted; (12) CRISPR prediction. By using CRISPRFinder software, CRISPRs can be recognized and we can obtain DRs and spacers.

3 SEQUENCING DATA STATISTIC

3.1 Solexa Sequencing Data

3.1.1 Method

There exists a certain amount of low quality data in raw data. In order to obtain more accurate and reliable results in subsequent bioinformatics analysis, the raw data will be treated.

The raw data treatment for 500 bp, 2,000 bp and 6,000 bp library of sample ADN Sec UMAF0158 includes the following steps:

(1) Read1 selects 1 bp- 90 bp, read2 selects 1 bp- 90 bp;

(2) Remove reads with a certain proportion of low quality (20) bases (40% as default, parameter setting at 36 bp);

(3)Remove reads with a certain proportion of Ns (10% as default, parameter setting at 9 bp);

(4) Remove adapter contamination (15bp overlap between adapter and reads as default, parameter setting at 15 bp);

(5) Remove duplication contamination.

The above processes are applied to read 1 and read 2 synchronously. After that, generally 10%-20% of the data is eliminated for small insert size reads. Because the duplication rate is high in large insert size reads, much more data are eliminated and there is no certain proportion.

3.1.2 Result

Data storage catalog: 00.Cleandata.

Table 3.1 shows statistical results after data treatment. The distribution of base percentage and quality along reads in data filtering are shown in Figure 3.1

Table 3.1 Reads data statistics

Sample Name	Insert Size (bp)	Raw Data (Mb)	GC Raw (%)	Low Quality (%)	N (%)	Adapter (%)	Duplication (%)	Clean Data (Mb)	GC Clean (%)
ADN Sec UMAF0158	507	1,336	(59.11, 59.27)	5.12	0.007	0.88	1.56	1,200	(59.04, 59.05)
	2,228	1,312	(58.79, 59.10)	3.19	0.002	1.98	0.75	1,200	(58.84, 58.90)
	6,321	1,352	(58.72, 59.11)	4.19	0.002	1.81	2.29	1,200	(58.93, 58.96)

Note: The columns from the left to the right refers to: sample name, insert size, raw data size, raw data GC content, the proportion of removed low quality data in raw data, the proportion of removed N in raw data, the proportion of removed adapter in raw data ,the proportion of removed duplication sequence size in raw data, usable data quantity after data filtration and GC content after data filtration.

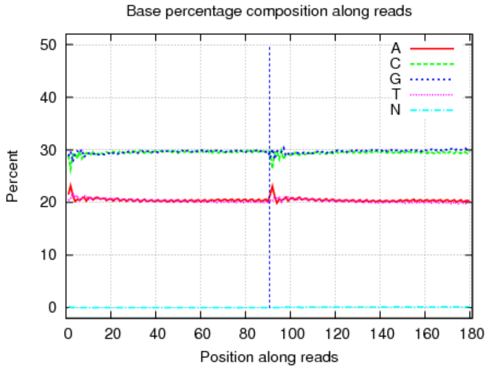

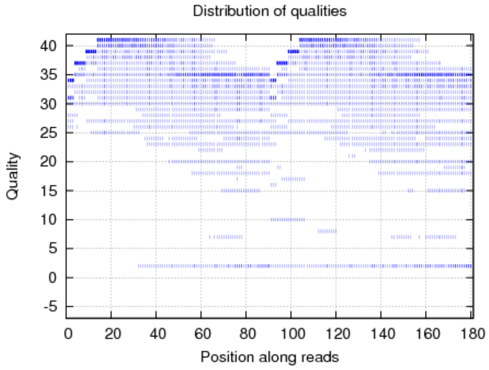

Figure 3.1.1.a 500bp

Figure 3.1.1.b 500bp

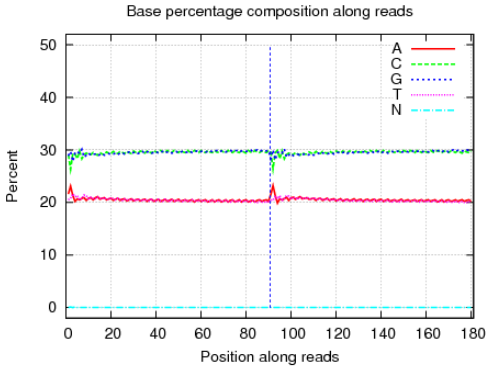

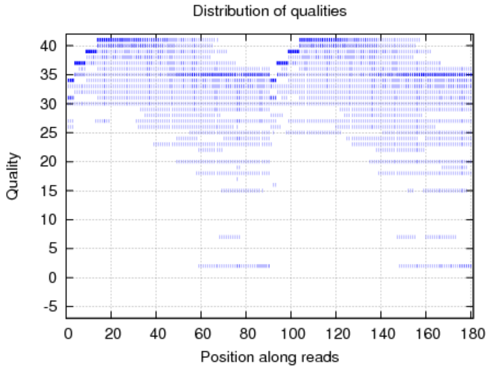

Figure 3.1.1.c 500 bp

Figure 3.1.1.d 500 bp

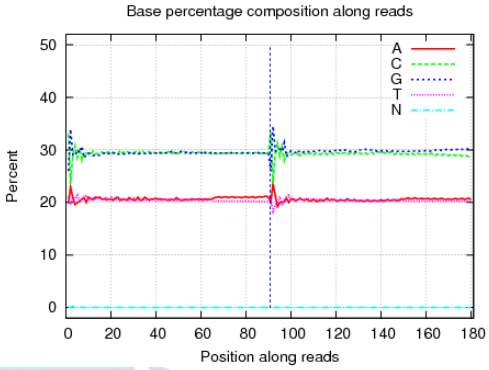

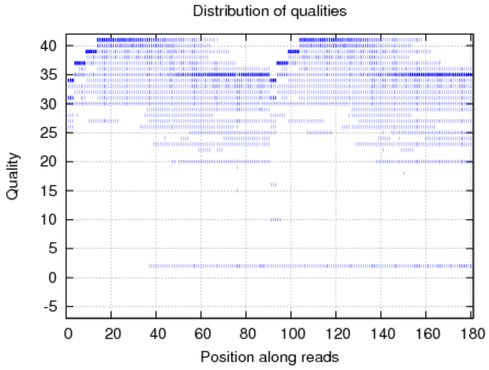

Figure 3.1.2.a 2,000bp

Figure 3.1.2.b 2,000bp

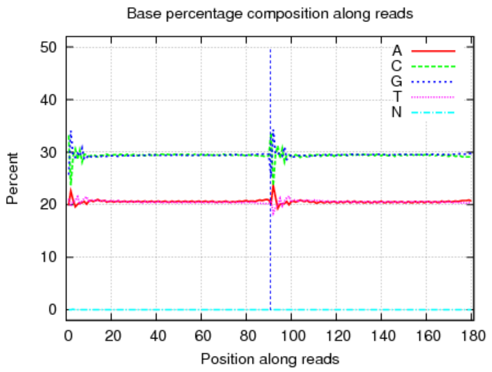

Figure 3.1.2.c 2,000 bp

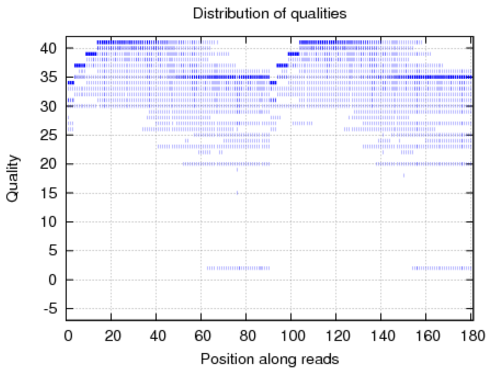

Figure 3.1.2.d 2,000 bp

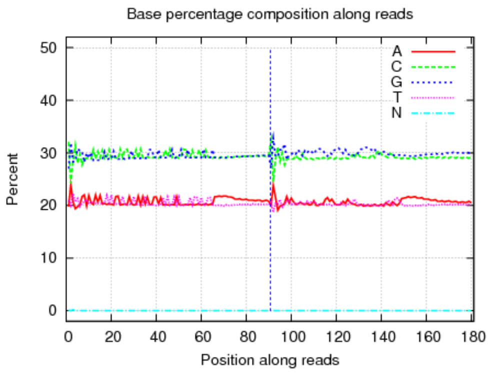

Figure 3.1.3.a 6,000bp

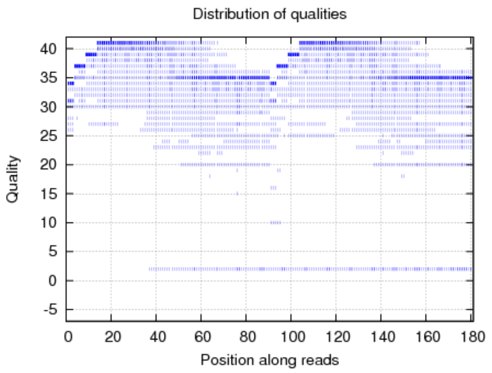

Figure 3.1.3.b 6,000bp

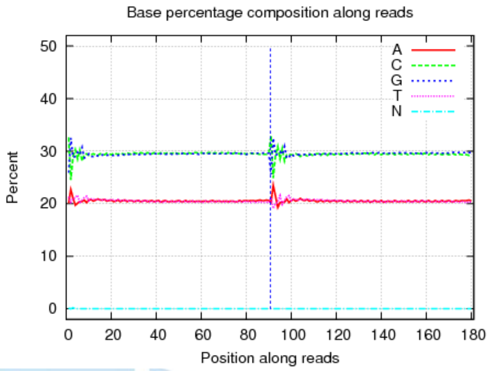

Figure 3.1.3.c 6,000 bp

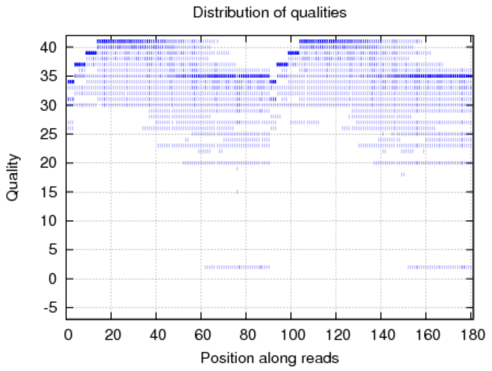

Figure 3.1.3.d 6,000 bp

Figure 3.1 Quality control. a) Base percentage distribution along reads before data filtering; b)

Distribution of qualities along reads before data filtering; c) Base percentage distribution along reads after data filtering ; d) Distribution of qualities along reads after data filtering.

4 GENOME SURVEY RESULT

4.1 Assembly Result

We apply SOAPdenovo (website: http://soap.genomics.org.cn/soapdenovo.html version:1.05) to assemble reads with insert size of 500 bp. Table 4.1 shows the optimal assembly results.

Table 4.1 Genome survey assembly result statistics

	Scaffold	Contig
**Total Number (#)**	20	245
**Total Length (bp)**	5,828,910	5,818,434
**Gap Length (bp)**	10,476	0
**Average Length (bp)**	291,446	23,749
**N50 (bp)**	930,427	42,536
**N90 (bp)**	213,886	13,138
**Max Length (bp)**	1,606,660	156,104
**Min Length (bp)**	620	122
**Sequence GC (%)**	59.32	59.32

Note: The second column refers to the Scaffolds longer than 500bp and the third column is the statistic of Contig got by breaking scaffold (>=500bp) from the second column with N.

4.2 Survey Assessment Result

Table 4.2 Genome survey assessment result statistics

	Standard	Result
**Genome Size (M)**	≤10	5.8
**GC (%)**	30-65	Raw Data：(59.57;59.64)<br>Clean Data：(58.90;59.01)<br>Assembly：59.32
**Scaffold Number**	≤100	20
**Contig Number**	≤1,000	245
**Repeat (%)**	≤10	-
**Chromosome and Plasmid Number**	≤4	1
**Inhomogeneous Sequence**	No	No

5 COMPLETE MAP ASSEMBLY ANALYSIS

5.1 Method

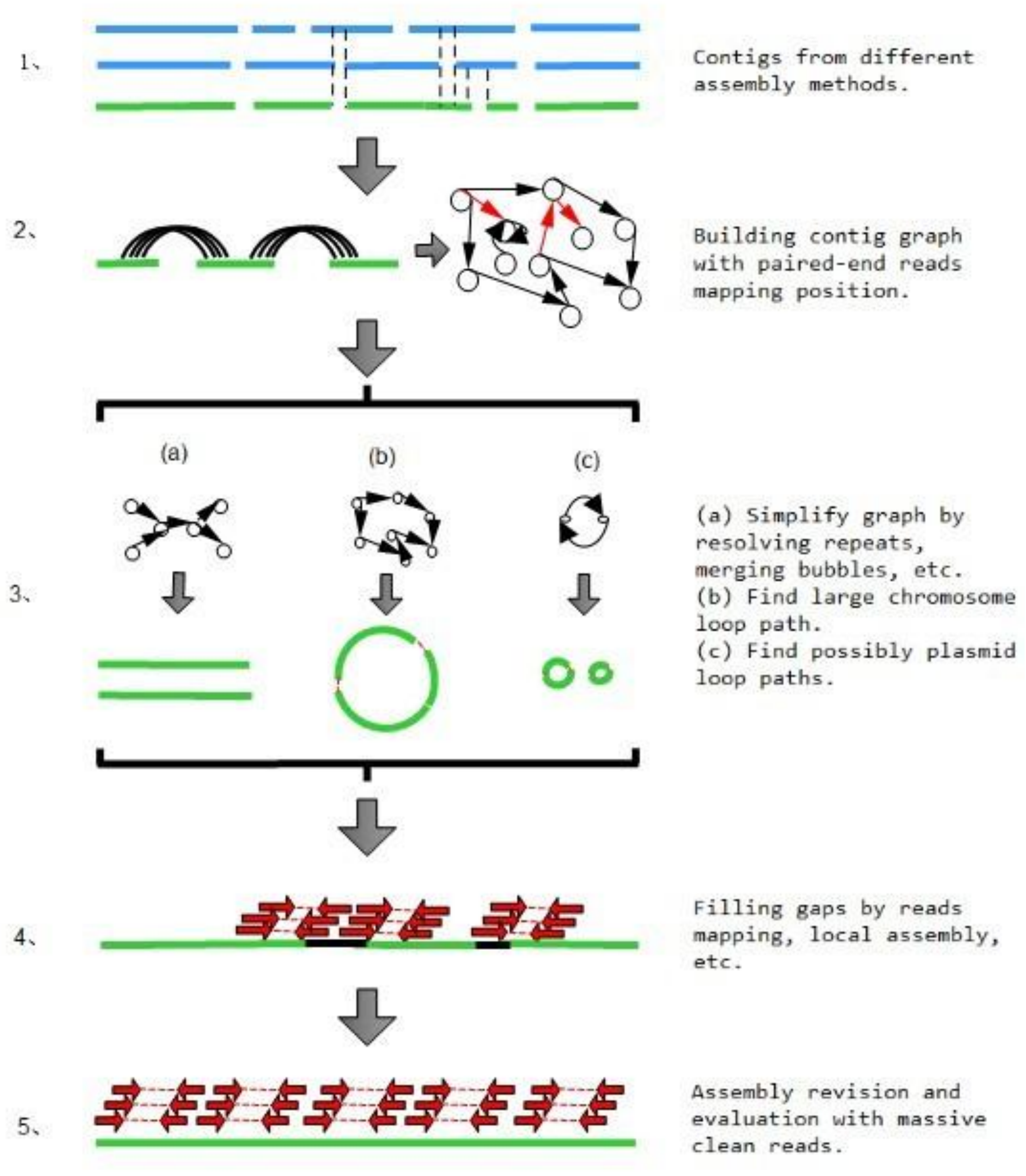

Figure 5.1 Advanced assembly schematic graph

(1) Preliminary assembly. Multiple assembly softwares are used to assemble treated

reads data. Afterwards, all assembly results are combined to construct much longer sequence and then to establish frame sequence.

(2) Construct Super-scaffold to ascertain position relationship of frame sequence. The potential position relationship of each frame sequence will be obtained through the analysis of mapping information of reads with paired-end relationship. The assembly principle is shown in Figure 5-1.

(3) The graph can be resolved according to some conditions and then the plasmid and circled genome can be obtained. Forwards, partial relationship complex situation will also be treated. Closely related reference sequence will do a great favor to the complete map assembly. The frame sequence can be ordered and positioned through the alignment between reference sequence and frame sequence. Further more, partial amendment will be performed through mapping reads with paired-end relationship to frame sequence.

(4) Local assembly and gap closure will be performed with paired-end reads locating in gaps. For highly complex region, PCR gap closure will be performed to obtain a sequence without outer gap.

(5) Error correction. SOAPaligner/soap2 software will be applied for error correction (website: http://soap.genomics.org.cn/soapaligner.html; version 2.21). Reads are mapped to sequence; mapping information are accounted and finally single base and local proofreading will be performed for assembly results.

5.2 Assembly Result before PCR

Assembly results statistics information is shown in Table 5.1. Assembly contain one chromosome (5,788,803bp) and another plasmid (62,961bp) circled respectively.

Table 5.1 Assembly results statistics

	Scaffold	Contig
**Total Number (#)**	2	4
**Total Length (bp)**	5,851,764	5,850,976
**Gap Length (bp)**	788	0
**Average Length (bp)**	2,925,882	1,462,744
**N50 (bp)**	5,788,803	1,997,991
**N90 (bp)**	5,788,803	1,207,479
**Max Length (bp)**	5,788,803	2,582,545
**Min Length (bp)**	62,961	62,961
**Sequence GC (%)**	59.28	59.28

6 PCR FILL GAP and VERIFICATION

6.1 Method

A certain length of sequence flanking low reliability region is chosen as PCR primer sequence for bilateral amplification. If the reads from both sides can form overlap, the PCR experiment for the verified region is successful. Afterwards， on the basis of the comparison between the sequence obtained by PCR product sequencing and the sequence of the low coverage region, the accuracy of low coverage region can be verified.

The complex sequence or inconvenient experiment condition will cause the failure of PCR amplification or that of sequencing. Therefore, the results of the verification of this process may include correctness, fault and unverified region.

When PCR sequencing results is different from the sequence of low reliability, the low reliability sequence will be replaced by PCR sequencing results.

PCR sequencing sketch map is shown in Figure 6.1.

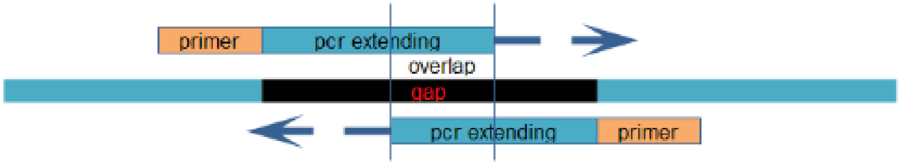

Figure 6.1 PCR sketch map

PCR primer is designed in the range of 1 Kbp flanking low reliability sequence*. The criteria for PCR primer designation include high randomness, non adenine at 3’end, low mismatch rate, no hairpin structure, no dimer, adequate annealing temperature and suitable primer length.

*PCR region is 1kbp at the ends of the region without gap and low coverage region. The two PCR regions will be combined into one PCR region if the two PCR regions’ distance shorter than 1kbp.

6.2 Result

Assembly results statistics before first cycle PCR and verification are shown in Table 6.1.

Table 6.1 Assembly results statistics before first cycle PCR and verification

**Sample**	ADN Sec UMAF0158
**Scaffold Number (#)**	2
**Total Length (bp)**	5,851,764
**Contig Number (#)**	4
**Gap Number (#)**	4
**Low Coverage Number (#)**	5

Gap number contains 2 inner gaps and 2 outer gaps.

Low reliability region and inner gap of assembly results are chosen to PCR and the statistics result are shown in Table 6.2.

Table 6.2 PCR and verification information statistics

**PCR Times**	**Total region (#)**	**Confirmed Region (#)**	**Unconfirmed Region (#)**
**First**	9	4	5
**Second**	5	4	1
**Third**	1	0	1

Assembly result statistics after three circle PCR verification are shown in Table 6.3.

Table 6.3 Assembly results statistics after PCR and verification

	Scaffold	Contig
**Total Number (#)**	2	2
**Total Length (bp)**	5,850,990	5,850,990
**Gap Length (bp)**	0	0
**Average Length (bp)**	2,925,495	2,925,495
**N50 (bp)**	5,787,986	5,787,986
**N90 (bp)**	5,787,986	5,787,986
**Max Length (bp)**	5,787,986	5,787,986
**Min Length (bp)**	63,004	63,004
**Sequence GC (%)**	59.28	59.28

7 ASSEMBLY QUALITY CONTROL

7.1 Method

600X clean data are aligned with the assembly results to account ISIZE (Insert Size) of each pair of reads. After removal of data whose ISIZE are shorter than 100bp or longer than 10K, the left data are ordered from small to big and the data between 2% to 98% are considered as pairing-eligible ISIZE. The alignment results are divided into three categories: mapped Paired-end Reads (PE Reads), unmapped Single-End Reads (SE Reads) and unmapped Reads (Unmapping Reads).

Reads use rate. Reads use rate refers to the ratio of mapped reads number to the total number reads ,Since there exists a certain degree of error rate in sequencing, this value will be less than 1. It can be judged empirically that under the condition that the sequencing error rate is 5%, reads use rate higher than 95% is normal.

Single base QC (Quality Control): The coverage is accounted for each base of PE Reads. The sites with depth less than 5 are considered as underproof sites. Single base QC pass rate refers to the ratio of number of sites passing through the quality control to the total sites number. The calculation formula is as follows:

Single Base Quality = (# of Depth>=5 Bases) / (# of Total Bases)

Structuredness QC: The alignment information statistics are performed for each base (Figure 3-1). The mapped reads are divided into Backward Buncheach base (r each base (s. The sites with depth less than 5 are considerate assembly results should conform the following conditions:

1. The depth of Backward Bunch and Forward Bunch should be higher than 3X*;

2. The pairing ISIZE of Backward Bunch and Forward Bunch should distribute in appropriate range;

3. The alignment position of all the mapped reads should meet a certain degree of randomness;

4. The depth of Single Bunch is approximately equal to zero.

(*Under ideal condition, the depth of both Backward Bunch and Forward Bunch should be equal to 1/2 of expectant depth. However, because of the strain genome feature and the impact of experimental condition, not all sites can attain expected effect. Consequently, under the precondition that the accounted lowest depth of single base is 6X, the depth of both Backward Bunch and Forward Bunch should be higher than 3X.)

On the basis of the conditions above, the structruedness QC (quality control) can be performed for each base. Structuredness QC refers to the ratio of the number of site passing through the quality control to the total site number. The calculation formula is as follows:

Structure Base Quality = (# of QC-passed Bases) / (# of Total Bases)

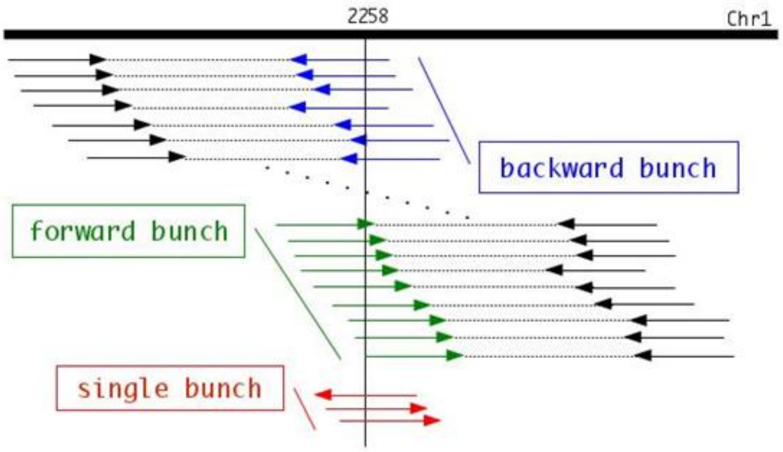

Figure 7.1 Structuredness quality control sketch diagram

7.2 Result

Quality control result is shown in Table 7.1.

Table 7-1 Quality result statistics

	Result	Reference Standard	Conclusion
**Single Base Quality (%)**	1.00	0.98	pass
**Structure Base Quality (%)**	0.99	0.98	pass
**Reads Usage Percent (%)**	0.99	0.95	pass

8 RESULT FEEDBACK

8.1 Assembly Result Statistics

After the assembly process above, the sequence has been assembled into complete map. Please refer to 01.Assembly/*.seq. The statistics results are shown in Table 8.1.

Table 8.1 Sequence information statistics

	Genome	Plasmid	Total
**Number (#)**	1	1	2
**Total Length (bp)**	5,787,986	63,004	5,850,990
**Average Length (bp)**	5,787,986	63,004	--
**GC (%)**	59.33	54.58	59.28
**Low Coverage Number (#)**	1	0	1

Note: Total refers to the sum of genome and plasmid.

Low Coverage information pleases refer to 01.Assembly/Low.coverage.list.

8.2 Repeat Sequence Statistics

From the analysis above, the length and GC content of target sequence can be obtained; the existence of heterogeneous sequence and the number of chromosome and that of plasmid can be judged. In the next step, the exact repeat sequence content can be calculated.

Method: By Blast software, sequence is aligned with itself and the regions of repeat sequence will be counted. The parameter is set as -e 1e-10. The identity value of sequence with alignment length no less than 50bp is no less than 90; the identity value of sequence with alignment length belonging to [25, 50) bp is equal to 100. If there

exist(s) plasmid(s), plasmid(s) and chromosomes will impact reciprocally. Therefore, the statistics of repeat sequence needs considering the content of both plasmid(s) and chromosome.

The statistics results are shown in Table 8.2.

Table 8.2 Repeat sequence statistics

	**Sequence**
**Repeat Area Number (#)**	305
**Repeat Ratio (%)**	1.2863
**Max Length (bp)**	5,806
**Min Length (bp)**	39
**Average Length (bp)**	246

10 DATA DOWNLOADING

Download

Host: http://cdts.genomics.org.cn/

ID: XXX

Password: XXX

Decompress the files

All the documents have been compressed under linux environment as *.gz, which can be decompressed by the following methods:

Unix/Linux user: gzip -d *.gz

Windows user: winRAR

Mac user: Shell：gzip -d *.gz

FTP directory structure

```
|-- Large project number_subproject code/
|       |--Sample name/
|       |       |--00.Cleandata/
|       |       |--01.Assembly/
|       |       |--02.PCR/
```

11 CONTACT US

Service Hotline: 400-706-6615

Customer Service: customer@genomics.com.cn

Technical Support: tech@genomics.com.cn

Complaint Hotline: 010-80481175(Beijing) 0755-25273291(Shenzhen)
