# Supplementary material for: Bioinformatics Analysis of the Complete Genome Sequence of the Mango Tree Pathogen Pseudomonas syringae pv. syringae UMAF0158 Reveals Traits Relevant to Virulence and Epiphytic Lifestyle
Source: PLoS One. 2015 Aug 27;10(8):e0136101. doi: 10.1371/journal.pone.0136101 (PMC4551802; doi:10.1371/journal.pone.0136101)
Supplement: S1 Table — (DOCX) [file pone.0136101.s007.docx]

**Table S1.** Accession numbers and references of genome sequences corresponding to 26 *Pseudomonas* strains used in this study.

| **Strain** | **Accession Number** | **Reference** |
| --- | --- | --- |
| *Pseudomonas syringae* 642 | NZ_ADGB00000000 | Clarke et al., 2010 |
| *Pseudomonas syringae aesculi* 0893 23 | NZ_AEAD00000000 | Baltrus et al., 2011 |
| *Pseudomonas syringae avellanae* ISPaVe037 | NZ_AKCK00000000 | O'Brien et al., 2012 |
| *Pseudomonas syringae* B64 | NZ_ANZF00000000 | Dudnik et al., 2013 |
| *Pseudomonas syringae* B728a | NC_007005 | Feil et al., 2005 |
| *Pseudomonas syringae* BRIP34876 | NZ_AMXK00000000 | Gardiner et al., 2013 |
| *Pseudomonas syringae* BRIP34881 | NZ_AMXL00000000 | Gardiner et al., 2013 |
| *Pseudomonas syringae* BRIP39023 | NZ_AMZX00000000 | Gardiner et al., 2013 |
| *Pseudomonas syringae* Cit 7 | NZ_AEAJ00000000 | Baltrus et al., 2011 |
| *Pseudomonas syringae glycinea* B076 | NZ_AEGG00000000 | Qi et al., 2011 |
| *Pseudomonas syringae glycinea* race 4 | NZ_AEGH00000000 | Qi et al., 2011 |
| *Pseudomonas syringae japonica* M301072 | NZ_AEAH00000000 | Baltrus et al., 2011 |
| *Pseudomonas syringae lachrymans* M302278 | NZ_AEAM00000000 | Baltrus et al., 2011 |
| *Pseudomonas syringae maculicola* ES4326 | NZ_AEAK00000000 | Baltrus et al., 2011 |
| *Pseudomonas syringae mori* 301020 | NZ_AEAG00000000 | Baltrus et al., 2011 |
| *Pseudomonas syringae morsprunorum* M302280 | NZ_AEAE00000000 | Baltrus et al., 2011 |
| *Pseudomonas syringae oryzae* 1 6 | NZ_ABZR00000000 | Reinhardt et al., 2009 |
| *Pseudomonas syringae phaseolicola* 1448A | NC_005773  NC_007274  NC_007275 | Joardar et al., 2005 |
| *Pseudomonas syringae tabaci* ATCC 11528 | NZ_AEAP00000000 | Baltrus et al., 2011 |
| *Pseudomonas syringae tomato* DC3000 | NC_004578  NC_004632  NC_004633 | Buell et al., 2003 |
| *Pseudomonas syringae tomato* K40 | NZ_ADFY00000000 | Cai et al., 2011 |
| *Pseudomonas syringae tomato* Max13 | NZ_ADFZ00000000 | Cai et al., 2011 |
| *Pseudomonas syringae tomato* NCPPB 1108 | NZ_ADGA00000000 | Cai et al., 2011 |
| *Pseudomonas syringae tomato* T1 | NZ_ABSM00000000 | Almeida et al., 2009 |
| *Pseudomonas savastanoi* NCPPB 3335 | NZ_ADMI00000000 | Rodríguez-Palenzuela et al., 2010 |
| *Pseudomonas syringae* UMAF0158 | CP005970  CP005971 | This work |
| *Pseudomonas fluorescens* Pf-5 | NC_004129 | Loper et al., 2007 |

**References**

Almeida NF, Yan S, Lindeberg M, Studholme DJ, Schneider DJ, Condon B, et al. A draft genome sequence of *Pseudomonas syringae* pv. *tomato* T1 reveals a type III effector repertoire significantly divergent from that of *Pseudomonas syringae* pv. *tomato* DC3000. Mol Plant Microbe Interact. 2009; 22: 52-62.

Baltrus DA, Nishimura MT, Romanchuk A, Chang JH, Mukhtar MS, Cherkis K, et al. Dynamic evolution of pathogenicity revealed by sequencing and comparative genomics of 19 *Pseudomonas syringae* isolates. PLoS Pathog. 2011; 7: e1002132.

Cai R, Lewis J, Yan S, Liu H, Clarke CR, Campanile F, et al. The plant pathogen *Pseudomonas syringae* pv. *tomato* is genetically monomorphic and under strong selection to evade tomato immunity. PLoS Pathog. 2011; 7: e1002130.

Clarke CR, Cai R, Studholme DJ, Guttman DS, Vinatzer BA. *Pseudomonas syringae* strains naturally lacking the classical *P. syringae hrp/hrc* Locus are common leaf colonizers equipped with an atypical type III secretion system. Mol Plant Microbe Interact. 2010; 23: 198-210.

Dudnik A, Dudler R. Non contiguous-finished genome sequence of *Pseudomonas syringae* pathovar *syringae* strain B64 isolated from wheat. Stand Genomic Sci. 2013; 8: 420-429.

Gardiner DM, Stiller J, Covarelli L, Lindeberg M, Shivas RG, Manners JM. Genome Sequences of *Pseudomonas* spp. Isolated from Cereal Crops. Genome Announc. 2013; 1(3).

Loper JE, Kobayashi DY, Paulsen IT. The Genomic Sequence of *Pseudomonas fluorescens* Pf-5: Insights Into Biological Control. Phytopathology 2007; 97: 233-238.

O'Brien HE, Thakur S, Gong Y, Fung P, Zhang J, Yuan L, et al. Extensive remodeling of the *Pseudomonas syringae* pv. *avellanae* type III secretome associated with two independent host shifts onto hazelnut. BMC Microbiol. 2012; 12: 141.

Qi M, Wang D, Bradley CA, Zhao Y. Genome sequence analyses of *Pseudomonas savastanoi* pv. *glycinea* and subtractive hybridization-based comparative genomics with nine pseudomonads. PLoS One 2011; 6: e16451.

Reinhardt JA, Baltrus DA, Nishimura MT, Jeck WR, Jones CD, Dangl JL. De novo assembly using low-coverage short read sequence data from the rice pathogen *Pseudomonas syringae* pv. *oryzae*. Genome Res. 2009; 19: 294-305.
