# Supplementary material for: Bioinformatics Analysis of the Complete Genome Sequence of the Mango Tree Pathogen Pseudomonas syringae pv. syringae UMAF0158 Reveals Traits Relevant to Virulence and Epiphytic Lifestyle
Source: PLoS One. 2015 Aug 27;10(8):e0136101. doi: 10.1371/journal.pone.0136101 (PMC4551802; doi:10.1371/journal.pone.0136101)
Supplement: S3 Table — (DOC) [file pone.0136101.s009.doc]

**Table S3.** Primers used in the construction of TTSS mutants of *Pseudomonas syringae* pv. syringae UMAF0158.

| TTSS | Name | Sequence | Size (bp) | Genes deleted | Size (bp) |
| --- | --- | --- | --- | --- | --- |
| *Mutagenesis primers* | |  |  |  |  |
| *rhc* | 1_TTSSII_for | ATGACGACTCCGAATAGGG | 476 | *rhcJ/hyp/rhcL/rhcNpartial* | 2518 |
|  | A_TTSSII_rev | CCCTATAGTGAGTCAAGCTTGCAGCGTTTGGACATGAT |  |  |  |
|  | B_TTSSII_for | AAGCTTGACTCACTATAGGGCAGTATCGTGCGTAACAGC | 458 |  |  |
|  | 2_TTSSII_rev | CGAGAATGGAACGGGTTT |  |  |  |
| *hrp* | 1_158_for | CAGTCTGGCGGCGTTATC | 509 | *hrpL* | 639 |
|  | A_158_rev | CCCTATAGTGAGTCAAGCTTCAAGCCCTATAGCGATAAC |  |  |  |
|  | B_158_for | AAGCTTGACTCACTATAGGGGTCTGGAACCAACTCGCA | 738 |  |  |
|  | 2_158_rev | GTCCTTGTCGGCATTGTC |  |  |  |
| *Mutagenesis checking primers* | |  |  |  |  |
| rhc_check_for | | GCCGAGCCTTATGCCTAT | 2981 | *rhcJ/hyp/rhcL/rhcNpartial* |  |
| rhc_check_rev | | TTCGCCCAACAGACCTTC |  |  |  |
| hrpL_check_for | | GTTCCTGTTGCTCGCCAATC | 1748 | *hrpL* |  |
| hrpL_check_rev | | CTCGGCGCTGACATTGCC |  |  |  |
| Km_for | | GAATGAACTGCAGGACG | 415 | *Km* |  |
| Km_rev | | ATTCGGCAAGCAGGCATC |  |  |  |
