# Supplementary material for: Bioinformatics Analysis of the Complete Genome Sequence of the Mango Tree Pathogen Pseudomonas syringae pv. syringae UMAF0158 Reveals Traits Relevant to Virulence and Epiphytic Lifestyle
Source: PLoS One. 2015 Aug 27;10(8):e0136101. doi: 10.1371/journal.pone.0136101 (PMC4551802; doi:10.1371/journal.pone.0136101)
Supplement: S4 Table — ORFs were first predicted and annotated by the NCBI Prokaryotic Genome Annotation Pipeline. Then, annotation was manually curated. ORFs highlighted in grey correspond to T4SS components (VirB, VirD), replication protein A and ultraviolet light resistance proteins A and B. (DOC) [file pone.0136101.s010.doc]

**Table S4.** Predicted ORF in *Pseudomonas syringae* pv. syringae plasmid (pPSS158, Gene Bank accession number CP005971). ORFs were first predicted and annotated by the NCBI Prokaryotic Genome Annotation Pipeline. Then, annotation was manually curated. ORFs highlighted in grey correspond to T4SS components (VirB, VirD), replication protein A and ultraviolet light resistance proteins A and B.

| **Locus Tag** | **Location** | **Strand** | **Length AA** | **Product** |
| --- | --- | --- | --- | --- |
| PSYRMG_25705 | 719..895 | - | 58 | hypothetical protein |
| PSYRMG_25710 | 987..1520 | + | 177 | transcriptional regulator |
| PSYRMG_25715 | 1609..1851 | + | 80 | hypothetical protein |
| PSYRMG_25720 | 1864..2571 | + | 235 | VirB1 |
| PSYRMG_25725 | 2568..2906 | + | 112 | VirB2 |
| PSYRMG_25730 | 2919..3407 | + | 162 | VirB3 |
| PSYRMG_25735 | 3256..5808 | + | 850 | VirB4 |
| PSYRMG_25740 | 5805..6491 | + | 228 | VirB5 |
| PSYRMG_25745 | 6524..6877 | + | 117 | hypothetical protein |
| PSYRMG_25750 | 6888..7820 | + | 310 | VirB6 |
| PSYRMG_25751* | 7888..8190 | + | 100 | VirB7 |
| PSYRMG_25755 | 8228..9016 | + | 262 | VirB8 |
| PSYRMG_25760 | 9006..9815 | + | 269 | VirB9 |
| PSYRMG_25765 | 9802..11172 | + | 456 | VirB10 |
| PSYRMG_25770 | 11182..12213 | + | 343 | VirB11 |
| PSYRMG_25775 | 12223..12579 | + | 118 | hypothetical protein |
| PSYRMG_25780 | 12706..12936 | + | 76 | hypothetical protein |
| PSYRMG_25785 | 12936..14588 | + | 550 | VirD4 |
| PSYRMG_25790 | 14630..14992 | + | 120 | killer protein |
| PSYRMG_25795 | 15020..17185 | + | 721 | DNA topoisomerase III |
| PSYRMG_25800 | 17213..17977 | + | 254 | hypothetical protein |
| PSYRMG_25805 | 18042..18647 | + | 201 | single-stranded DNA-binding protein |
| PSYRMG_25810 | 18954..19697 | + | 247 | stability protein |
| PSYRMG_25815 | 19694..20266 | + | 190 | hypothetical protein |
| PSYRMG_25820 | 20293..20715 | - | 140 | hypothetical protein |
| PSYRMG_25825 | 21057..21782 | + | 241 | relaxosome component |
| PSYRMG_25826* | 21772..25515 | + | 1247 | Relaxase/Orf54 |
| PSYRMG_25830 | 21985..22218 | - | 77 | hypothetical protein |
| PSYRMG_25835 | 22411..22695 | - | 94 | hypothetical protein |
| PSYRMG_25840 | 22769..23191 | + | 140 | hypothetical protein |
| PSYRMG_25845 | 23491..25515 | + | 674 | hypothetical protein |
| PSYRMG_25850 | 25645..26295 | + | 216 | ParA family protein |
| PSYRMG_25855 | 26286..26639 | + | 117 | hypothetical protein |
| PSYRMG_25860 | 27197..28498 | + | 433 | replication protein A |
| PSYRMG_25865 | 29288..29575 | - | 95 | hypothetical protein |
| PSYRMG_25870 | 29626..29913 | - | 95 | hypothetical protein |
| PSYRMG_25875 | 30182..31138 | + | 318 | 6-hydroxy-3-succinoylpyridine hydroxylase NicB |
| PSYRMG_25880 | 31339..31764 | + | 141 | ultraviolet light resistance protein A |
| PSYRMG_25885 | 31742..33040 | + | 432 | ultraviolet light resistance protein B |
| PSYRMG_25890 | 33579..33986 | + | 135 | hypothetical protein |
| PSYRMG_25895 | 34324..34524 | + | 66 | hypothetical protein |
| PSYRMG_25900 | 34638..35288 | + | 216 | plasmid partitioning protein ParA |
| PSYRMG_25905 | 35278..35565 | + | 95 | hypothetical protein |
| PSYRMG_25910 | 35758..35976 | + | 72 | hypothetical protein |
| PSYRMG_25915 | 35973..36512 | + | 179 | hypothetical protein |
| PSYRMG_25920 | 36788..37429 | + | 213 | hypothetical protein |
| PSYRMG_25925 | 37817..39163 | + | 448 | hypothetical protein |
| PSYRMG_25930 | 39230..40135 | - | 301 | LysR transcriptional regulator |
| PSYRMG_25935 | 40229..40855 | + | 208 | dimethylmenaquinone methyltransferase/h. prot. |
| PSYRMG_25940 | 40872..41741 | + | 289 | hypothetical protein |
| PSYRMG_25945 | 42093..42596 | - | 167 | hypothetical protein |
| PSYRMG_25950 | 42766..43464 | - | 232 | hypothetical protein |
| PSYRMG_25955 | 43662..43952 | + | 96 | hypothetical protein |
| PSYRMG_25960 | 44047..44370 | - | 107 | hypothetical protein |
| PSYRMG_25965 | 44532..44771 | + | 79 | hypothetical protein |
| PSYRMG_25970 | 44808..45059 | + | 83 | ArdR protein |
| PSYRMG_25975 | 45180..45488 | + | 102 | hypothetical protein |
| PSYRMG_25980 | 45553..45699 | - | 48 | hypothetical protein |
| PSYRMG_25985 | 45954..48599 | + | 881 | hypothetical protein |
| PSYRMG_25990 | 48633..48881 | + | 82 | hypothetical protein |
| PSYRMG_25995 | 48954..49232 | + | 92 | hypothetical protein |
| PSYRMG_26000 | 49250..51286 | - | 678 | chemotaxis protein |
| PSYRMG_26005 | 51453..51629 | - | 58 | hypothetical protein |
| PSYRMG_26010 | 52584..52844 | + | 86 | hypothetical protein |
| PSYRMG_26015 | 52937..53071 | + | 44 | hypothetical protein |
| PSYRMG_26020 | 53072..55936 | - | 954 | PAS/PAC sensor histidine kinase |
| PSYRMG_26025 | 56649..57164 | + | 171 | diguanylate cyclase |
| PSYRMG_26030 | 57367..58212 | - | 281 | LuxR family transcriptional regulator |
| PSYRMG_26035 | 58502..59461 | - | 319 | integrase |
| PSYRMG_26040 | 59679..60179 | + | 166 | Hcp1 family type VI secretion system effector |
| PSYRMG_26045 | 61096..61869 | - | 257 | LuxR family transcriptional regulator |

* ORFs not predicted by NCBI Prokaryotic Genome Annotation Pipeline. Locus tags of these genes are not yet definitive.
