# Supplementary material for: Bioinformatics Analysis of the Complete Genome Sequence of the Mango Tree Pathogen Pseudomonas syringae pv. syringae UMAF0158 Reveals Traits Relevant to Virulence and Epiphytic Lifestyle
Source: PLoS One. 2015 Aug 27;10(8):e0136101. doi: 10.1371/journal.pone.0136101 (PMC4551802; doi:10.1371/journal.pone.0136101)
Supplement: S5 Table — Gray shading indicates genes which are present in B728a (E-value < 1e-10). (DOCX) [file pone.0136101.s011.docx]

| **Region** | **Product** | **Start**  **(bp)** | **End**  **(bp)** | **Length (aa)** | **Strand** |
| --- | --- | --- | --- | --- | --- |
| **Region 1:**  **248304-257525 (9221 bp)** |  |  |  |  |  |
| **Locus Tag** |  |  |  |  |  |
| PSYRMG_01130 | LuxR family transcriptional regulator | 248304 | 248639 | 111 | - |
| PSYRMG_01135 | hypothetical protein | 249307 | 249804 | 165 | - |
| PSYRMG_01140 | repressor | 249908 | 250603 | 231 | - |
| PSYRMG_01145 | Cro/Cl family transcriptional regulator | 250687 | 250926 | 79 | + |
| PSYRMG_01150 | hypothetical protein | 251167 | 251964 | 265 | + |
| PSYRMG_01155 | DNA replication protein DnaC | 251954 | 252748 | 264 | + |
| PSYRMG_01160 | hypothetical protein | 252750 | 252944 | 64 | + |
| PSYRMG_01165 | hypothetical protein | 252941 | 253237 | 98 | + |
| PSYRMG_01170 | VRR-NUC domain-containing protein | 253234 | 253665 | 143 | + |
| PSYRMG_01175 | integrase | 253662 | 254942 | 426 | + |
| PSYRMG_01180 | hypothetical protein | 254959 | 255177 | 72 | + |
| PSYRMG_01185 | hypothetical protein | 255174 | 255419 | 81 | + |
| PSYRMG_01190 | hypothetical protein | 255416 | 255772 | 118 | + |
| PSYRMG_01195 | antitermination protein Q | 255775 | 256161 | 128 | + |
| PSYRMG_01200 | hypothetical protein | 256326 | 256610 | 94 | + |
| PSYRMG_01205 | hypothetical protein | 256728 | 257525 | 265 | + |
| **Region 2:**  **258593-272300 (13707 bp)** |  |  |  |  |  |
| **Locus Tag** |  |  |  |  |  |
| PSYRMG_01225 | hypothetical protein | 258593 | 258733 | 46 | + |
| PSYRMG_01230 | hypothetical protein | 258737 | 259087 | 116 | + |
| PSYRMG_01235 | hypothetical protein | 259077 | 259253 | 58 | + |
| PSYRMG_01240 | HNH endonuclease | 259308 | 259703 | 131 | + |
| PSYRMG_01245 | terminase | 259872 | 260357 | 161 | + |
| PSYRMG_01250 | terminase | 260358 | 262091 | 577 | + |
| PSYRMG_01255 | hypothetical protein | 262088 | 262252 | 54 | + |
| PSYRMG_01260 | histone H1 | 262245 | 263555 | 436 | + |
| PSYRMG_01265 | peptidase | 263559 | 264401 | 280 | + |
| PSYRMG_01270 | capsid protein | 264413 | 265798 | 461 | + |
| PSYRMG_01275 | hypothetical protein | 265850 | 266266 | 138 | + |
| PSYRMG_01280 | hypothetical protein | 266270 | 266743 | 157 | + |
| PSYRMG_01285 | head-tail adaptor protein | 266743 | 267081 | 112 | + |
| PSYRMG_01290 | hypothetical protein | 267074 | 267559 | 161 | + |
| PSYRMG_01295 | hypothetical protein | 267559 | 267927 | 122 | + |
| PSYRMG_01300 | major tail subunit | 267991 | 268491 | 166 | + |
| PSYRMG_01305 | lambda gpG-like protein | 268501 | 268938 | 145 | + |
| PSYRMG_01310 | hypothetical protein | 268995 | 269141 | 48 | + |
| PSYRMG_01315 | tail tape measure protein | 269189 | 271762 | 857 | + |
| PSYRMG_01320 | hypothetical protein | 271762 | 272106 | 114 | + |
| PSYRMG_01325 | hypothetical protein | 272103 | 272300 | 65 | + |
| **Region 3:**  **519318-537403 (18085 bp)** |  |  |  |  |  |
| **Locus Tag** |  |  |  |  |  |
| PSYRMG_02460 | hypothetical protein | 519318 | 522788 | 1156 | - |
| PSYRMG_02465 | hypothetical protein | 523423 | 523584 | 53 | + |
| PSYRMG_02470 | SMAD/FHA domain protein | 523773 | 524663 | 296 | + |
| PSYRMG_02475 | hypothetical protein | 524660 | 525019 | 119 | + |
| PSYRMG_02480 | type III secretion component | 525016 | 525354 | 112 | + |
| PSYRMG_02485 | Harpin secretion protein hrpI | 525351 | 527498 | 715 | + |
| PSYRMG_02490 | secretin | 527495 | 528811 | 438 | + |
| PSYRMG_02495 | hypothetical protein | 528824 | 529240 | 138 | + |
| PSYRMG_02500 | hypothetical protein | 529243 | 530202 | 319 | - |
| PSYRMG_02505 | type III secretion protein | 530201 | 530812 | 203 | + |
| PSYRMG_02510 | hypothetical protein | 530833 | 531051 | 72 | + |
| PSYRMG_02515 | hypothetical protein | 531157 | 531492 | 111 | + |
| PSYRMG_02520 | type III secretion protein | 531496 | 532275 | 259 | + |
| PSYRMG_02525 | type III secretion protein | 532272 | 532895 | 207 | + |
| PSYRMG_02530 | type III secretion protein | 532888 | 533484 | 198 | + |
| PSYRMG_02535 | ATP synthase | 533481 | 534809 | 442 | + |
| PSYRMG_02540 | hypothetical protein | 534918 | 535421 | 167 | + |
| PSYRMG_02545 | type III secretion protein | 535418 | 536476 | 352 | + |
| PSYRMG_02550 | flagellar biosynthesis protein FliP | 536473 | 537129 | 218 | + |
| PSYRMG_02555 | EscS | 537134 | 537403 | 89 | + |
| **Region 4:**  **996632-1011913 (15281 bp)** |  |  |  |  |  |
| **Locus Tag** |  |  |  |  |  |
| PSYRMG_03980...PSYRMG_04060  (17 genes) | hypothetical protein |  |  |  |  |
| **Region 5:**  **1133844-1143487 (9643 bp)** |  |  |  |  |  |
| **Locus Tag** |  |  |  |  |  |
| PSYRMG_04645...PSYRMG_04690  (10 genes) | hypothetical protein |  |  |  |  |
| PSYRMG_04695 | hypothetical protein | 1138610 | 1139044 | 144 | + |
| PSYRMG_04700 | hypothetical protein | 1139127 | 1139783 | 218 | - |
| PSYRMG_04705 | hypothetical protein | 1140350 | 1140607 | 85 | + |
| PSYRMG_04710...PSYRMG_04730  (5 genes) | hypothetical protein |  |  |  |  |
| PSYRMG_04735 | integrase | 1142215 | 1143090 | 291 | + |
| PSYRMG_04740 | hypothetical protein | 1143143 | 1143487 | 114 | + |
| **Region 6:**  **1357822 - 1378644 (20822 bp)** |  |  |  |  |  |
| **Locus Tag** |  |  |  |  |  |
| PSYRMG_05730...PSYRMG_05745  (4 genes) | hypothetical protein |  |  |  |  |
| PSYRMG_05750 | ShlB family hemolysin secretion/activation protein | 1376935 | 1378644 | 569 | - |
| **Region 7:**  **2201422 - 2221045 (19623 bp)** |  |  |  |  |  |
| **Locus Tag** |  |  |  |  |  |
| PSYRMG_09545 | integrase | 2201422 | 2202582 | 386 | + |
| PSYRMG_09550 | integrase | 2202569 | 2204476 | 635 | + |
| PSYRMG_09555 | integrase | 2204521 | 2206398 | 625 | + |
| PSYRMG_09560...PSYRMG_09600  (9 genes) | hypothetical protein |  |  |  |  |
| PSYRMG_09605 | transcriptional regulator | 2213307 | 2213675 | 122 | - |
| PSYRMG_09610, PSYRMG_09615  (2 genes) | hypothetical protein |  |  |  |  |
| PSYRMG_09620 | hypothetical protein | 2214681 | 2215067 | 128 | + |
| PSYRMG_09625 | hypothetical protein | 2215190 | 2215900 | 236 | + |
| PSYRMG_09630 | hypothetical protein | 2217780 | 2218073 | 97 | + |
| PSYRMG_09630...PSYRMG_09650  (5 genes) | hypothetical protein |  |  |  |  |
| **Region 8:**  **2233554 - 2244999 (11445 bp)** |  |  |  |  |  |
| **Locus Tag** |  |  |  |  |  |
| PSYRMG_09715...PSYRMG_09780  (14 genes) | hypothetical protein |  |  |  |  |
| PSYRMG_09785 | thiamin biosynthesis protein | 2244996 | 2246141 | 381 | - |
| PSYRMG_09790 | hypothetical protein | 2246158 | 2246907 | 249 | - |
| PSYRMG_09795 | DNA-binding protein | 2247120 | 2247491 | 123 | + |
| PSYRMG_09800 | peptidase | 2247506 | 2248372 | 288 | + |
| PSYRMG_09805...PSYRMG_09840  (8 genes) | hypothetical protein |  |  |  |  |
| PSYRMG_09845 | mechanosensitive ion channel protein | 2255655 | 2256269 | 204 | + |
| PSYRMG_09850, PSYRMG_09855  (2 genes) | hypothetical protein |  |  |  |  |
| PSYRMG_09860 | hypothetical protein | 2257718 | 2259910 | 730 | + |
| PSYRMG_09865, PSYRMG_09870  (2 genes) | hypothetical protein |  |  |  |  |
| PSYRMG_09875 | antitoxin | 2262230 | 2262424 | 64 | + |
| **Region 9:**  **2324538 - 2330589 (6051 bp)** |  |  |  |  |  |
| **Locus Tag** |  |  |  |  |  |
| PSYRMG_10110 | transporter | 2324538 | 2325485 | 315 | - |
| PSYRMG_10115 | amidinotransferase | 2325501 | 2326598 | 365 | - |
| PSYRMG_10120 | biotin carboxylase | 2326614 | 2327777 | 387 | - |
| PSYRMG_10125 | hypothetical protein | 2327774 | 2328985 | 403 | - |
| PSYRMG_10130 | oxidoreductase | 2329000 | 2329710 | 236 | - |
| PSYRMG_10135 | hypothetical protein | 2329912 | 2330589 | 225 | - |
| **Region 10:**  **2710838 - 2721328 (10490 bp)** |  |  |  |  |  |
| **Locus Tag** |  |  |  |  |  |
| PSYRMG_11895...PSYRMG_11940  (10 genes) | hypothetical protein |  |  |  |  |
| **Region 11:**  **3032080 - 3050678 (18598 bp)** |  |  |  |  |  |
| **Locus Tag** |  |  |  |  |  |
| PSYRMG_13360...PSYRMG_13370  (3 genes) | hypothetical protein |  |  |  |  |
| PSYRMG_13375 | XRE family transcriptional regulator | 3035822 | 3036112 | 96 | - |
| PSYRMG_13380...PSYRMG_13425  (10 genes) | hypothetical protein |  |  |  |  |
| **Region 12:**  **4684145 - 4696189 (12044 bp)** |  |  |  |  |  |
| **Locus Tag** |  |  |  |  |  |
| PSYRMG_20805 | cell division protein FtsQ | 4684145 | 4685284 | 379 | + |
| PSYRMG_20810 | cellulose synthase | 4685281 | 4687500 | 739 | + |
| PSYRMG_20815 | divalent ion tolerance protein CutA | 4687515 | 4689770 | 751 | + |
| PSYRMG_20820 | endo-1,4-D-glucanase | 4689774 | 4690985 | 403 | + |
| PSYRMG_20825 | cellulose synthase | 4690961 | 4694836 | 1291 | + |
| PSYRMG_20830 | cell division protein FtsQ | 4694853 | 4695518 | 221 | + |
| PSYRMG_20835 | cell division protein FtsQ | 4695515 | 4696189 | 224 | + |
| **Region 13:**  **5668482 - 5685647 (17165 bp)** |  |  |  |  |  |
| **Locus Tag** |  |  |  |  |  |
| PSYRMG_25225...PSYRMG_25325  (21 genes) | hypothetical protein |  |  |  |  |
