# Supplementary material for: Bioinformatics Analysis of the Complete Genome Sequence of the Mango Tree Pathogen Pseudomonas syringae pv. syringae UMAF0158 Reveals Traits Relevant to Virulence and Epiphytic Lifestyle
Source: PLoS One. 2015 Aug 27;10(8):e0136101. doi: 10.1371/journal.pone.0136101 (PMC4551802; doi:10.1371/journal.pone.0136101)
Supplement: S6 Table — Gray shading indicates genes which are present in UMAF0158 (E-value < 1e-10). (DOCX) [file pone.0136101.s012.docx]

| **Region** | **Product** | **Start**  **(bp)** | **End**  **(bp)** | **Length**  **(aa)** | **Strand** |
| --- | --- | --- | --- | --- | --- |
| **Region 1:**  **102358 - 116873 (14515 bp)** |  |  |  |  |  |
| **Locus Tag** |  |  |  |  |  |
| YP_233205.1 | flavoprotein monooxygenase | 102358 | 103761 | 467 | - |
| YP_233206.1 | hypothetical protein | 103896 | 104600 | 234 | + |
| YP_233207.1 | transposase IS4 | 104951 | 106369 | 472 | - |
| YP_233208.1 | transposase IS4 | 106580 | 107563 | 327 | - |
| YP_233209.1...YP_233212.1  (4 genes) | hypothetical protein |  |  |  |  |
| YP_233213.1 | hypothetical protein | 112987 | 113487 | 166 | - |
| YP_233214.1...YP_233216.1  (3 genes) | hypothetical protein |  |  |  |  |
| **Region 2:**  **838293 - 843261 (4968 bp)** |  |  |  |  |  |
| **Locus Tag** |  |  |  |  |  |
| YP_233835.1 | relaxase | 838293 | 839249 | 318 | - |
| YP_233836.1 | hypothetical protein | 839680 | 840123 | 147 | - |
| YP_233837.1 | peptidase S24, S26A and S26B | 840487 | 840921 | 144 | + |
| YP_233838.1 | umuC protein | 840908 | 841153 | 81 | + |
| YP_233839.1 | transmembrane protein | 841367 | 841780 | 137 | - |
| YP_233840.1 | type III effector protein AvrRpm1 | 842575 | 843261 | 228 | - |
| **Region 3:**  **1614863 - 1658265 (43402 bp)** |  |  |  |  |  |
| **Locus Tag** |  |  |  |  |  |
| YP_234515.1...YP_234521.1  (7 genes) | hypothetical protein |  |  |  |  |
| YP_234522.1 | C-5 cytosine-specific DNA methylase | 1621535 | 1622950 | 471 | - |
| YP_234523.1 | antibiotic biosynthesis monooxygenase | 1623523 | 1623834 | 103 | - |
| YP_234524.1 | regulatory protein LysR | 1623981 | 1624916 | 311 | - |
| YP_234525.1 | hypothetical protein | 1624983 | 1626230 | 415 | + |
| YP_234526.1 | regulatory protein LysR | 1626243 | 1627124 | 293 | + |
| YP_234527.1 | Beta-lactamase | 1628265 | 1629257 | 330 | + |
| YP_234528.1 | DSBA oxidoreductase | 1629396 | 1630082 | 228 | - |
| YP_234529.1...YP_234546.1  (18 genes) | hypothetical protein |  |  |  |  |
| YP_234547.1 | DNA polymerase, beta-like region | 1646308 | 1647093 | 261 | + |
| YP_234548.1, YP_234549.1  (2 genes) | hypothetical protein |  |  |  |  |
| YP_234550.1 | N-acetyltransferase GCN5 | 1649070 | 1649609 | 179 | + |
| YP_234551.1...YP_234560.1  (10 genes) | hypothetical protein |  |  |  |  |
| **Region 4:**  **1672580 - 1678069 (5489 bp)** |  |  |  |  |  |
| **Locus Tag** |  |  |  |  |  |
| YP_234571.1...YP_234573.1  (3 genes) | hypothetical protein |  |  |  |  |
| YP_234574.1 | Cl- channel, voltage gated | 1673655 | 1675001 | 448 | - |
| YP_234575.1 | hypothetical protein | 1675250 | 1675573 | 107 | - |
| YP_234576.1 | bile acid:sodium symporter | 1675584 | 1676327 | 247 | - |
| YP_234577.1 | hypothetical protein | 1677287 | 1677565 | 92 | - |
| YP_234578.1 | hypothetical protein | 1677692 | 1678069 | 125 | + |
| **Region 5:1692009 - 1713389 (21380 bp)** |  |  |  |  |  |
| **Locus Tag** |  |  |  |  |  |
| YP_234593.1 | hypothetical protein | 1692009 | 1692284 | 91 | + |
| YP_234594.1 | cation efflux protein | 1692284 | 1693237 | 317 | + |
| YP_234595.1 | hypothetical protein | 1693576 | 1694016 | 146 | + |
| YP_234596.1 | Phage integrase:Phage integrase, N-terminal SAM-like | 1694121 | 1695080 | 319 | + |
| YP_234597.1 | PilM protein | 1695639 | 1696079 | 146 | - |
| YP_234598.1 | prepilin | 1696111 | 1697517 | 468 | - |
| YP_234599.1 | type II secretion system protein E | 1697514 | 1698446 | 310 | - |
| YP_234600.1 | prepilin | 1698449 | 1698973 | 174 | - |
| YP_234601.1 | type II secretion system protein | 1699012 | 1700103 | 363 | - |
| YP_234602.1 | type II secretion system protein E | 1700093 | 1701724 | 543 | - |
| YP_234603.1, YP_234604.1  (2 genes) | hypothetical protein |  |  |  |  |
| YP_234605.1 | type II and III secretion system protein | 1703673 | 1705409 | 578 | - |
| YP_234606.1 | hypothetical protein | 1705406 | 1706539 | 377 | - |
| YP_234607.1 | SecC motif-containing protein | 1706691 | 1708157 | 488 | - |
| YP_234608.1 | SNF2-like protein | 1708268 | 1710220 | 650 | - |
| YP_234609.1 | hypothetical protein | 1710223 | 1712109 | 628 | - |
| YP_234610.1 | single-stranded DNA-binding protein | 1712925 | 1713389 | 154 | - |
| **Region 6:**  **3182993 - 3199241 (16248 bp)** |  |  |  |  |  |
| **Locus Tag** |  |  |  |  |  |
| YP_235700.1 | hypothetical protein | 3182993 | 3184252 | 419 | - |
| YP_235701.1 | lipoprotein | 3184308 | 3184808 | 166 | - |
| YP_235702.1 | lipoprotein | 3184816 | 3185553 | 245 | - |
| YP_235703.1, YP_235704.1  (2 genes) | hypothetical protein |  |  |  |  |
| YP_235705.1 | ABC transporter | 3188330 | 3189022 | 230 | - |
| YP_235706.1 | von Willebrand factor, type A | 3189024 | 3191015 | 663 | - |
| YP_235707.1, YP_235708.1  (2 genes) | hypothetical protein |  |  |  |  |
| YP_235709.1 | virulence protein SrfB | 3194740 | 3197874 | 1044 | - |
| YP_235710.1 | hypothetical protein | 3197886 | 3199241 | 451 | - |
| **Region 7:**  **3207940 - 3242247 (34307 bp)** |  |  |  |  |  |
| **Locus Tag** |  |  |  |  |  |
| YP_235717.1 | NUDIX hydrolase | 3207940 | 3208386 | 148 | + |
| YP_235718.1, YP_235719.1  (2 genes) | hypothetical protein |  |  |  |  |
| YP_235720.1 | hypothetical protein | 3210976 | 3212205 | 409 | - |
| YP_235721.1 | hypothetical protein | 3212729 | 3214660 | 643 | + |
| YP_235722.1 | hypothetical protein | 3215082 | 3215459 | 125 | - |
| YP_235723.1...YP_235729.1  (7 genes) | hypothetical protein |  |  |  |  |
| YP_235730.1 | ISPsy8, transposase OrfA | 3224311 | 3224826 | 171 | + |
| YP_235731.1 | integrase catalytic subunit | 3224856 | 3225662 | 268 | + |
| YP_235732.1 | nitroreductase | 3225794 | 3226615 | 273 | + |
| YP_235733.1...YP_235741.1  (9 genes) | hypothetical protein |  |  |  |  |
| YP_235742.1 | phenazine biosynthesis PhzC/PhzF protein | 3235541 | 3236047 | 168 | + |
| YP_235743.1 | NUDIX hydrolase | 3236226 | 3236753 | 175 | - |
| YP_235744.1 | transposase Tn3 | 3236917 | 3239802 | 961 | - |
| YP_235745.1 | helix-turn-helix, Fis-type | 3239928 | 3240542 | 204 | + |
| YP_235746.1 | aminoglycoside phosphotransferase | 3240608 | 3241411 | 267 | + |
| YP_235747.1 | aminoglycoside/hydroxyurea antibiotic resistance kinase | 3241417 | 3242247 | 276 | + |
| **Region 8:**  **3368488 - 3409506 (41018 bp)** |  |  |  |  |  |
| **Locus Tag** |  |  |  |  |  |
| YP_235858.1...YP_235868.1  (11 genes) | hypothetical protein |  |  |  |  |
| YP_235869.1 | Phage head morphogenesis protein, SPP1 gp7 | 3374929 | 3376017 | 362 | - |
| YP_235870.1...YP_235876.1  (7 genes) | hypothetical protein |  |  |  |  |
| YP_235877.1 | hypothetical protein | 3381323 | 3381553 | 76 | - |
| YP_235878.1 | hypothetical protein | 3381700 | 3382149 | 149 | + |
| YP_235879.1 | lipoprotein | 3382185 | 3382523 | 112 | - |
| YP_235880.1 | hypothetical protein | 3382523 | 3382894 | 123 | - |
| YP_235881.1, YP_235882.1  (2 genes) | hypothetical protein |  |  |  |  |
| YP_235883.1 | bacteriophage lambda NinG | 3384124 | 3384708 | 194 | - |
| YP_235884.1 | protein NinB | 3384708 | 3385106 | 132 | - |
| YP_235885.1...YP_235888.1  (4 genes) | hypothetical protein |  |  |  |  |
| YP_235889.1 | peptidase | 3388088 | 3388738 | 216 | + |
| YP_235890.1 | hypothetical protein | 3388751 | 3389023 | 90 | + |
| YP_235891.1...YP_235896.1  (6 genes) | hypothetical protein |  |  |  |  |
| YP_235897.1 | RecT protein | 3394228 | 3395115 | 295 | + |
| YP_235898.1, YP_235899.1  (2 genes) | hypothetical protein |  |  |  |  |
| YP_235900.1 | hypothetical protein | 3397214 | 3397597 | 127 | + |
| YP_235901.1 | hypothetical protein | 3398125 | 3398907 | 260 | - |
| YP_235902.1 | DNA methylase N-4/N-6 | 3399004 | 3400125 | 373 | + |
| YP_235903.1, YP_235904.1  (2 genes) | hypothetical protein |  |  |  |  |
| YP_235905.1 | C-5 cytosine-specific DNA methylase | 3401768 | 3403822 | 684 | + |
| YP_235906.1 | hypothetical protein | 3404194 | 3404508 | 104 | + |
| YP_235907.1 | hypothetical protein | 3404505 | 3405149 | 214 | + |
| YP_235908.1 | hypothetical protein | 3405489 | 3405950 | 153 | + |
| YP_235909.1 | Phage integrase:Phage integrase, N-terminal SAM-like | 3406247 | 3407281 | 344 | + |
| YP_235910.1 | hypothetical protein | 3409060 | 3409506 | 148 | + |
| **Region 9:**  **4526331 - 4542603 (16272 bp)** |  |  |  |  |  |
| **Locus Tag** |  |  |  |  |  |
| YP_236875.1...YP_236878.1  (4 genes) | hypothetical protein |  |  |  |  |
| YP_236879.1 | integrase catalytic subunit | 4531582 | 4532403 | 273 | - |
| YP_236880.1 | transposase IS3/IS911 | 4532460 | 4532759 | 99 | - |
| YP_236881.1 | type III effector HopAF1 | 4533648 | 4534502 | 284 | + |
| YP_236882.1 | N-acetyltransferase GCN5 | 4535275 | 4535808 | 177 | + |
| YP_236883.1 | hypothetical protein | 4535995 | 4538115 | 706 | + |
| YP_236884.1, YP_236885.1  (2 genes) | hypothetical protein |  |  |  |  |
| **Region 10:**  **5507043 - 5520779 (13736 bp)** |  |  |  |  |  |
| **Locus Tag** |  |  |  |  |  |
| YP_237711.1 | conjugal transfer protein | 5507043 | 5508014 | 323 | + |
| YP_237712.1 | plasmid stabilization system protein | 5508664 | 5509014 | 116 | - |
| YP_237713.1 | prevent-host-death protein | 5509027 | 5509275 | 82 | - |
| YP_237714.1 | Phage integrase:Phage integrase, N-terminal SAM-like | 5509622 | 5510575 | 317 | + |
| YP_237715.1, YP_237716.1  (2 genes) | hypothetical protein |  |  |  |  |
| YP_237717.1 | N-acetyltransferase GCN5 | 5511307 | 5511816 | 169 | - |
| YP_237718.1 | S-type Pyocin | 5513236 | 5515167 | 643 | + |
| YP_237719.1 | colicin immunity protein/pyocin immunity protein | 5515172 | 5515447 | 91 | + |
| YP_237720.1...YP_237723.1  (4 genes) | hypothetical protein |  |  |  |  |
| YP_237724.1 | type III effector HopAB1 | 5519229 | 5520779 | 516 | - |
